# Imaging non-classical mechanical responses of lipid membranes using molecular rotors†

**DOI:** 10.1039/d0sc05874b

**Published:** 2020-12-22

**Authors:** Miguel Páez-Pérez, Ismael López-Duarte, Aurimas Vyšniauskas, Nicholas J. Brooks, Marina K. Kuimova

**Affiliations:** MSRH, Department of Chemistry, Imperial College London Wood Lane London W12 0BZ UK m.kuimova@imperial.ac.uk n.brooks@imperial.ac.uk; Departamento de Química Orgánica, Universidad Autónoma de Madrid Cantoblanco 28049 Madrid Spain; Center of Physical Sciences and Technology Saulėtekio av. 3 Vilnius Lithuania

## Abstract

Lipid packing in cellular membranes has a direct effect on membrane tension and microviscosity, and plays a central role in cellular adaptation, homeostasis and disease. According to conventional mechanical descriptions, viscosity and tension are directly interconnected, with increased tension leading to decreased membrane microviscosity. However, the intricate molecular interactions that combine to build the structure and function of a cell membrane suggest a more complex relationship between these parameters. In this work, a viscosity-sensitive fluorophore (‘molecular rotor’) is used to map changes in microviscosity in model membranes under conditions of osmotic stress. Our results suggest that the relationship between membrane tension and microviscosity is strongly influenced by the bilayer's lipid composition. In particular, we show that the effects of increasing tension are minimised for membranes that exhibit liquid disordered (L_d_) – liquid ordered (L_o_) phase coexistence; while, surprisingly, membranes in pure gel and L_o_ phases exhibit a negative compressibility behaviour, *i.e.* they soften upon compression.

## Abstract 2

Viscosity-sensitive molecular rotors demonstrate that the non-classical mechanical behaviour of model lipid membranes is able to buffer external stress.

## Graphical abstract

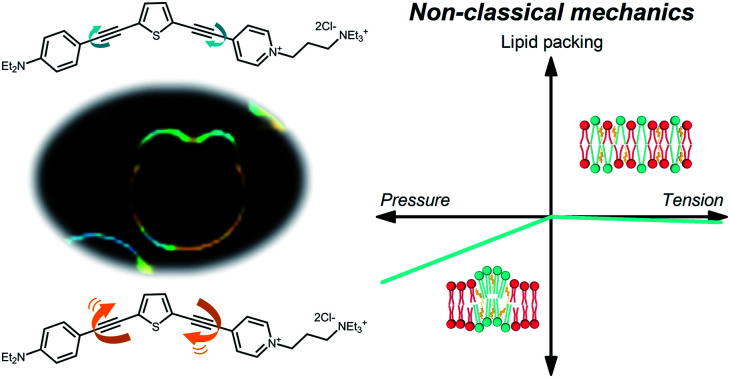

## Introduction

Cellular plasma membranes are known to play a crucial role in determining cell fate and behaviour. In addition to serving as a protective barrier between a cell and its surroundings, it also allows the cell to sense its environment. The plasma membrane is particularly suited to transduce biophysical stimuli such as the morphology and topology of its surroundings, external forces or electromagnetic fields;^1–3^ as well as to react to these cues. Examples of these responses include alteration of cell shape,^4^ density,^5^ mechanical^6,7^ and electrical^8^ properties. This has been proven to be crucial in a number of diseases such as cancer, malaria, sickle cell anaemia or atherosclerosis (AS).^9,10^ It has been therefore proposed that studying these diseases from a mechanical point of view, as an alternative to traditional biochemical-based approaches,^11–13^ may provide a more robust biophysical understanding of these conditions, leading to a better understanding of the disease, as well as diagnosis and treatment.

In the simplest case, the cell membrane could be modelled as a two-dimensional fluid lipid bilayer, embedding various proteins.^14^ Such bilayer can be characterized by its microviscosity *η*, which is proportional to the degree of lipid packing. Lipid packing is determined by the membrane tension, *σ*, the overall result of repulsive and attractive forces between lipid molecules.^15,16^ In steady state conditions *σ* tends to be minimised, which for membranes composed of a mixture of lipids may lead to the emergence of phase-separated domains. Although there is still some debate on the underlaying explanation for the heterogeneous organization of lipid membranes, the most commonly accepted reasons include reducing the hydrophobic mismatch between different lipids,^17–19^ van der Waals interactions between lipid molecules and entropy of the lipid acyl chains^20^ or local membrane curvature.^21,22^ The resulting lateral organisation of the plasma membrane is of great importance in signal transduction, as acknowledged in the ‘lipid raft’ hypothesis,^23^ which suggests that cholesterol-rich, more viscous microdomains of increased order in the cell membrane act as signalling and trafficking hubs.

However, the membrane's tension (and therefore viscosity) can change under the application of external stress, such as pressure,^24,25^ stretching^26,27^ or shearing.^28,29^ According to conventional mechanics, tensile efforts decrease lipid packing and membrane microviscosity, while compressive forces should have the opposite effect. Yet, several studies suggested that in practice a different behaviour is observed. For instance, while lipid packing was found to decrease under shear,^30,31^ the response to micropipette and osmotic tensioning of model membranes was heterogenous and depended on the lipid composition and the probing method.^32–34^ In fact, increased tension has been shown to trigger phase separation, to reorganize lipid domains and to determine their size and shape.^26,27,32,35,36^ On the other hand, pressure-induced phase separation has also been observed^37^ as well as an increase of the lipid packing of the L_d_ phase for single component membranes,^38^ whereas when L_d_ and L_o_ regions coexisted, packing of the more viscous L_o_ domains was increased instead.^24^ Furthermore, it has been proposed that solid gel domains may undergo pressure-induced softening,^39^ an unconventional mechanical response which may be a determinant step in the function of auditory hair cells.^40^ All in all, these results suggest there is a lipid-organization dependent response to external stress that warrants further studies.

Common ways to quantitatively evaluate membrane fluidity and microviscosity, such as fluorescence correlation spectroscopy,^41^ fluorescence recovery after photobleaching^42^ or single particle tracking,^43^ are not easily compatible with mapping large sample areas. Furthermore, their time resolution is limited, which prevents them from monitoring dynamic processes. On the other hand, environmentally sensitive fluorescent probes avoid these limitations. For example, Laurdan-based probes are frequently used to monitor lipid packing and phase by detecting changes in the bilayer's polarity, associated with accessibility to water.^31,44,45^ However, membrane microviscosity and polarity are not necessarily coupled.^46^ Molecular rotors have emerged as true viscosity-sensitive molecular probes which promise to overcome the issues outlined above by directly providing fast and quantitative spatiotemporally-resolved mapping of the lipid bilayer's microviscosity.^47–51^

The working principle of molecular rotors is based on the fact that the efficiency of the non-radiative decay in these fluorophores is coupled to the intramolecular motion of the rotor. Following excitation in less viscous environments, in which the intramolecular motion is not restricted, non-radiative decay becomes the predominant deactivation channel, leading to a decrease in the fluorescence quantum yield (*Φ*_f_, eqn (1))^50,52^ and lifetime (*τ*_f_, eqn (2))^50,53^ at lower viscosities, as expressed by the Förster–Hoffmann equation:^54^1
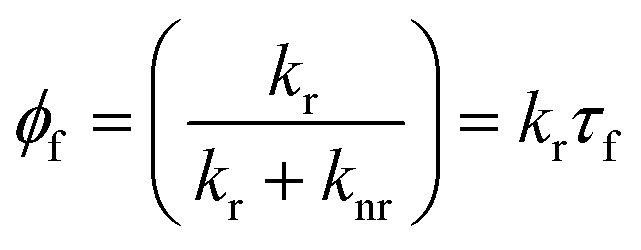

2 *τ*_f_ = *zη*^*α*^

where *k*_r_ and *k*_nr_ are radiative and non-radiative decay constants, *η* is viscosity, and *z* and *α* are constants. This relationship allows a direct calibration of molecular rotor responses to viscosity. Of note, eqn (2) uses fluorescence lifetime, which is independent of the probe's concentration and, hence, could be used even when the probe's uptake and concentration are unknown. Thus, molecular rotors have been successfully used to measure the micromechanical properties of model bilayers as large and giant unilamellar vesicles, LUVs and GUVs,^48,55,56^ as well as various membranes in prokaryotic^57^ and eukaryotic^58,59^ cells, *via* Fluorescence Lifetime Imaging Microscopy (FLIM). Furthermore, molecular rotors have enabled to quantify the viscosity changes of lipid bilayers under external forces such as flow-induced shear^60^ or hyper-gravity conditions.^61^ More recently, Colom *et al.* used a fluorescent tension probe with a similar working principle to molecular rotors to investigate the changes in lipid packing of tensed membranes, and found that tension was altered for phase-separated membranes while, surprisingly, no change was observed for single phase DOPC bilayers.^32^

**Fig. 1:**
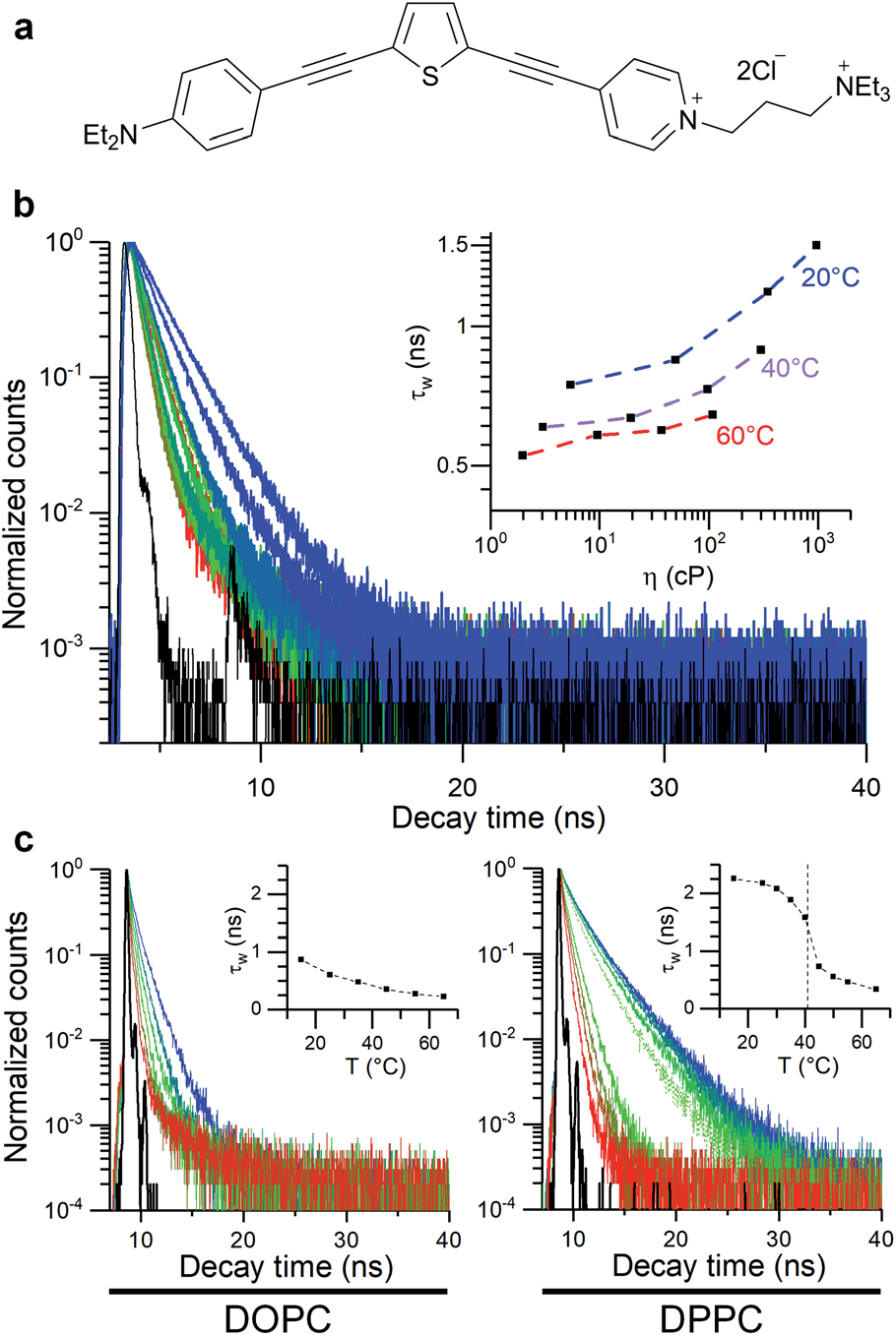
Molecular structure of the thiophene dye 1 used in this work (a) and its time-resolved decay traces in toluene/castor oil mixtures of variable viscosity (b) and in lipid bilayers (DOPC and DPPC LUVs), (c). Dashed line in (c) represents DPPC gel to liquid transition temperature.

In this work, thiophene-based molecular rotors (Fig. 1)^56,62^ are used to investigate how the bilayer composition affects the viscosity response of model membranes under osmotically induced stress. The ability of this probe to equally partition between the less viscous lipid disordered (L_d_) and more tightly packed lipid ordered (L_o_) phases^56^ allows to efficiently monitor the effect of membrane stress on both of these regions, *via* FLIM. This is particularly important, since this probe allows to directly study the L_o_ phase that makes up highly ordered membrane microdomains, the transduction hubs thought to play a key role in a number of diseases such as atherosclerosis.^28,44,63^

## Results and discussion

### Thiophene-based molecular rotors as viscosity sensors

The thiophene-based dye 1, Fig. 1, has been previously shown to retain in cell plasma membranes of cultured mammalian cells and was suggested as a sensor of membrane potential.^62^ At the same time, dye 1 has been shown to have molecular rotor properties, displaying a reduced fluorescence lifetime upon the fluidification of lipid bilayers.^56^

In order to quantify viscosity sensitivity of 1 we measured the steady state and time-resolved fluorescence responses of the dye in castor oil/toluene calibration mixtures. While these mixtures have similar polarity to DOPC-based lipid membranes,^56^ their viscosity can be varied between 2–1000 cP by varying their composition and temperature. Furthermore, by utilising these mixtures, the temperature effect on the photophysical behaviour of these dyes can be directly investigated.^53^

Emission spectra and time-resolved decay traces of 1 were recorded, and the intensity-weighted average lifetime (*τ*_w_) was calculated for a biexponential fit according to:3
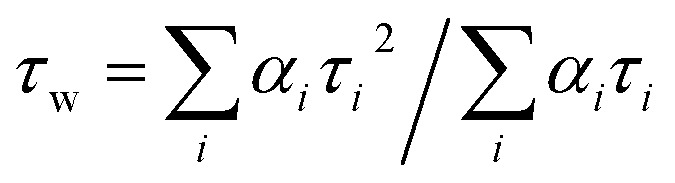
where *i* represents the decay components.

A log–log plot of *τ*_w_ against viscosity showed a clear increase in lifetime and fluorescence intensity with increased viscosity (Fig. 1 and S1†), together with a small temperature-dependent offset. The small offset may be due to the direct temperature sensitivity of 1 (Fig. 1).^52,53^ In any case, this data indicate that we must perform our measurements at a fixed temperature, to be able to trust the calibration values of our viscosity sensor.

We also studied the viscosity-sensitive behaviour of dye 2, where the acetylenic bridge of 1 is substituted for its olefinic counterpart (Fig. S2a†). The motivation for comparing dyes 1 and 2 came from the fact that both 1 and 2 localise in the plasma membranes of live cells and are suitable as second harmonic generation (SHG) microscopy probes, while 2 has a higher quantum yield, which is beneficial for imaging applications.^62^ However, our data indicates that 2 is not significantly sensitive to viscosity (Fig S2b–f†).

We used 1 incorporated in model membranes to record the membrane response to temperature variations. A temperature decrease results in a gradual increase in fluorescence lifetime of 1 incorporated into DOPC vesicles, corresponding to a smooth increase in microviscosity. Conversely, in a DPPC bilayer, a liquid-to-gel phase transition could be seen at 41 °C, by monitoring a change in the gradient of lifetime/temperature slope (Fig. 1). Furthermore, the increased membrane hydration upon fluidification^56,62,64^ is evidenced by a slight red shift in fluorescence spectra of 1 (Fig. S3c†). The microviscosity and polarity sensitivity of 1 was also confirmed in phase-separated GUVs composed by DOPC, DPPC and cholesterol (Fig. S4†). Overall, in these simple model membranes, 1 responds as expected from a viscosity-dependent molecular rotor.

### Effect of lipid composition on the stress response of LUVs

As mentioned above, the stress response of lipid bilayers appears to strongly depend on lipid composition. We set out to use molecular rotor-based FLIM to directly monitor microviscosity of lipid membranes upon compression and stretching, induced by osmotic gradients, including in phase-separated membranes. The use of a single probe that equally partitions to lipid domains of varied packing^56^ would enable us to follow trends in microviscosity quantitatively. The use of diffraction-limited fluorescence lifetime-based microscopy together with GUVs will also allow to monitor domain emergence and disappearance directly, as well as their microviscosity.

**Fig. 2:**
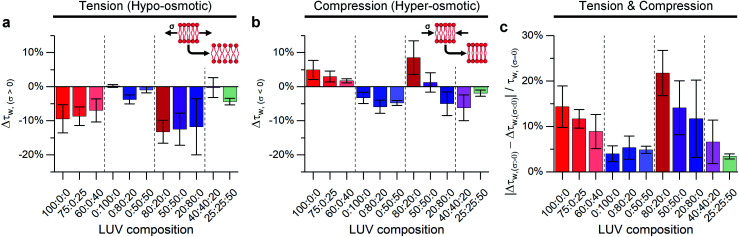
The effect of DOPC : DPPC : cholesterol ternary LUV composition on lifetime variations of 1 expressed as: Δ*τ*_w_ = (*τ*_w_/*τ*_w,*σ*=0_) − 1 compared at 23 °C to iso-osmotic conditions for (a) membrane tension (Δ*C* = 0.36 M, *σ* = 0.098 mN m^−1^); (b) membrane compression (Δ*C* = −1.08 M, *σ* = −0.063 mN m^−1^) and (c) difference between hypo- and hyper-osmotic lifetime divided by lifetime under iso-osmotic conditions. Inset shows the expected change in lipid packing for a membrane following classical mechanics. Bars show mean ± S.D (*n* = 3).

We compared lifetimes of 1 against the tension created by an osmotic gradient (isotropic stress, see ESI† for membrane tension calculation) in DOPC/DPPC/cholesterol membranes with varying percentages of each lipid (Fig. 2 and S5†). Two notably unexpected behaviours were observed: (i) negative compressibility of membranes in the solid gel and liquid-ordered phases and (ii) tension buffering *via* strain-hardening in membranes displaying L_o_/L_d_ phase coexistence.

### Negative compressibility of gel phase membranes

Initially, binary composition lipid bilayers containing either DOPC : cholesterol or DPPC : cholesterol were subjected to both tensile and compressive stress. Pure DOPC vesicles followed classical mechanics, decreasing their microviscosity under hypo-osmotic (*i.e.* increased tension due to water influx) and increasing microviscosity under hyper-osmotic (*i.e.* compression due to water efflux) conditions. However, by adding cholesterol, the resilience of the membrane properties to mechanical stress was increased, Fig. 2c. Cholesterol is known to insert in the L_d_ phase, filling free space created by the DOPC double bonds^65,66^ and increasing lipid ordering.^32,46,67^ This increased ordering is reflected in longer lifetimes recorded for 1 in the bilayers in the presence of cholesterol (Fig. S5†). In our experiments we see a significant reduction in the change of lifetime of 1 under osmotic shock in the presence of cholesterol (0% Chol > 25% > 40%, Fig. 2). Based on this data we hypothesise that cholesterol insertion could sterically resist DOPC chain compression. However, a higher amount of cholesterol was required for buffering hypo-osmotic induced tension; suggesting that a minimum cholesterol fraction is required to effectively rigidify the membrane against tensile stress, Fig. 2a.

In contrast to DOPC LUVs, DPPC LUVs remained unchanged under tension, Fig. 2a, probably owing to the stronger Van der Waals interactions between DPPC molecules in gel phase.^68^ On the contrary, bilayer compression resulted in a decrease of lifetime of 1, an unexpected result that suggests DPPC membranes become more laterally disordered and soften under pressure, a behaviour referred to as negative compressibility. Additional experiments were also performed to rule out possible artefacts due to a pressure-induced displacement of 1 out of the membrane^69^ (Fig. S6†).

Adding cholesterol to DPPC membranes leads to an increase in the lifetime of 1 (Fig. S5†), this is likely to be due to a local ordering effect of cholesterol^65,70^ or closer association between the rotor and the lipid's hydrocarbon tail.^56^ However, adding cholesterol did not alter the membrane's response to compression (Fig. 2), presumably because the lipid chain conformational order is not significantly reduced by cholesterol. On the other hand, DPPC : cholesterol LUVs became more fluid when subjected to tensile stress, leading to a behaviour akin to L_d_ phase of DOPC.

Our working hypothesis is that highly ordered lipid bilayers (*e.g.* gel phase of DPPC) soften with increased pressure, as previously predicted theoretically by Diggins IV *et al.*^39^ As opposed to DOPC, tightly packed gel-phase DPPC membranes cannot undergo further compaction due to the lack of free volume that was provided by DOPC unsaturation. Instead, the resulting excess lipid might create highly curved membrane protrusions.^71,72^ The likelihood of membrane buckling is discussed further in the ESI.†

**Fig. 3:**
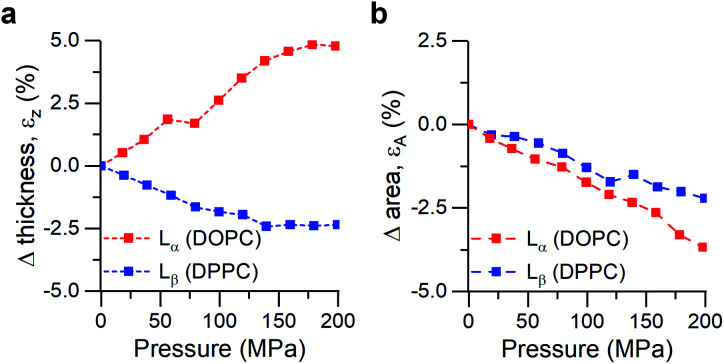
Calculated strains from SAXS/WAXS traces for DOPC and DPPC bilayers under pressure. (a) Strain normal to the membrane plane, defined as *ε*_z_ = Δ*d*_HH_/*d*_HH,0_, (b) Area strain of the membrane plane, defined as *ε*_A_ = Δ*A*/*A*_0_. See ESI† for detailed information.

We performed small- and wide-angle X-ray diffraction (SAXS/WAXS) experiments on DOPC and DPPC hydrated bilayer stacks under hydrostatic pressure (0–200 MPa) to further investigate this hypothesis (Fig. 3). Changes in the bilayer thickness were probed by SAXS, while WAXS was used to investigate changes in lipid packing density (*i.e.* area per lipid). Because the position of the diffraction peaks in the small angle region arises from both the bilayer thickness (*d*_HH_) and the water layer in between lamellae, we followed the procedure suggested by Rappolt *et al.*^73^ to estimate the actual bilayer thickness.

Increased pressure resulted in lateral compression of unsaturated DOPC bilayers, as evidenced by the negative area strain *ε*_A_ (Fig. 3b), which in turn caused a membrane thickening (positive *ε*_z_, Fig. 3a) of around 5%, in agreement with our previously published work.^74^ This negative linear compressibility normal to the bilayer surface is a result of the anisotropic structure of the oriented lipid molecules which have a relatively high bulk modulus but significantly higher lateral compressibility due to the ability of the hydrocarbon chains to reduce their conformational disorder and consequently straighten out.

Gel phase DPPC molecules also increased their packing, albeit to a lesser extent, as was predicted by their lower area compressibility module. WAXS traces showed that increased pressure caused the peak maxima to shift towards higher *q*-values (increased lipid packing, Fig. 3b), and to change from the characteristic “peak and hump” shape of the tilted gel L_β′_ phase to a broader peak (similar to that of a rippled P_β′_, Fig. S7†). This effect was reversible once the pressure was reduced.

It has been previously hypothesized that the ripple phase (whose corrugations could be thought as membrane buckles) contains regions of lower order,^75^ in accordance with our observations of negative compressibility using the molecular rotor 1. Furthermore, contrary to the fluid DOPC bilayers, increased pressure on DPPC bilayers resulted in an overall reduction in their thickness (Fig. 3a), in agreement with previous reports using reduced temperature^76,77^ (equivalent to an increase in pressure according to the Clapeyron equation^78^).

In fact, the decrease of both bilayer thickness and lipid area indicates a negative Poisson ratio^79^ for the gel membranes (see ESI†).^80^ Thus, both X-ray data and the molecular rotor-based fluorescence data corroborate our conclusion of negative compressibility of DPPC in the gel phase.

### L_o_/L_d_ mediated stress buffering

We investigated binary DOPC : DPPC mixtures, where the coexistence of solid and liquid disordered domains was expected. For these compositions, the response to tension was similar for all DOPC : DPPC ratios: in all cases decrease in membrane microviscosity was observed under tension, same as for pure DOPC LUVs. We hypothesise that despite significant amounts of DPPC present, the presence of even a small amount of unsaturated lipid molecules may be able to disrupt long-range order in the bilayer, allowing the membrane to stretch.

In contrast, the response to membrane compression gradually changed with increasing DPPC content, resulting in a lower increase in membrane microviscosity. Eventually, negative compressibility behaviour was observed for DOPC : DPPC ≤ 20 : 80. This might indicate that even low amounts of unsaturated lipid would buffer the compression forces before they are transmitted to the L_o_ phase.

Adding cholesterol turns the gel L_β_ to liquid ordered L_o_ phase, altering the membrane's behaviour. As shown in Fig. 2 LUVs with a 40 : 40 : 20 composition (which exhibit L_o_/L_d_ phase coexistence) have an almost null decrease in microviscosity when subjected to tensile efforts, while compression, instead, triggers a decrease in membrane microviscosity. Yet, if cholesterol content is further increased to 50% of total lipid composition, a smaller change in lipid packing is observed, which we interpret as an increased buffering of compressive forces.

In summary, when no cholesterol is present (50 : 50 : 0 DOPC : DPPC : cholesterol LUV composition), the solid-like DPPC domains are not able to participate in stress buffering and a DOPC-like response is observed. On the other hand, when cholesterol is present, both L_d_ and L_o_ phases coexist, and they can rearrange upon tractive or compressive stress application;^24,35^ minimising the effect of tractive stress. However, further increasing the amount of cholesterol further decreases the free space between lipid molecules, favouring buffering of compressive forces instead.

**Fig. 4:**
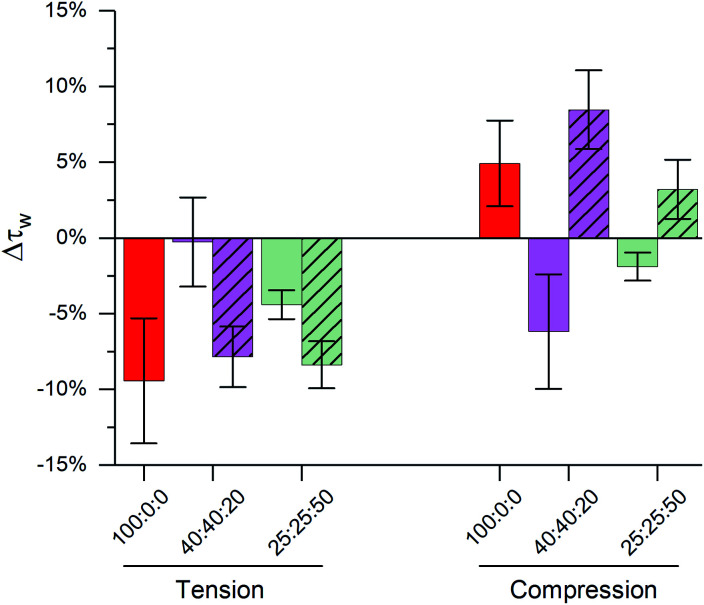
Effect of cholesterol content and temperature on buffering of membrane stress in ternary DOPC : DPPC : cholesterol LUVs. Clear bars represent data at *T* = 23 °C; dashed bars represent data from a single phase at *T* = 45 °C. Bars show mean ± S.D. (*n* = 3).

The importance of highly ordered membrane regions in stress buffering is highlighted in Fig. 4. At room temperature, when phase-separated membranes were formed, we observed tension buffering under tension and negative compressibility under compression. However, above the membrane's *T*_m_ (45 °C, *i.e.* single L_d_ phase) the response of both 40 : 40 : 20 and 25 : 25 : 50 LUVs was similar to DOPC ones (Fig. 4 and S8†). 40 : 40 : 20 LUVs were unable to buffer tension stress and the negative compressibility properties were lost for both 40 : 40 : 20 and 25 : 25 : 50 membranes. Even though these measurements were performed at a different temperature, we are confident in the trends observed, due to (i) these being relative measurements *vs.* zero tension and (ii) only a small effect of temperature on the photophysics of 1, Fig. 1.

### FLIM gives insight for L_o_/L_d_ stress buffering mechanism

In order to gain direct evidence on the domain's behaviour upon changes in osmotic pressure, the microviscosity changes for phase separated GUVs were examined *via* FLIM.

**Fig. 5:**
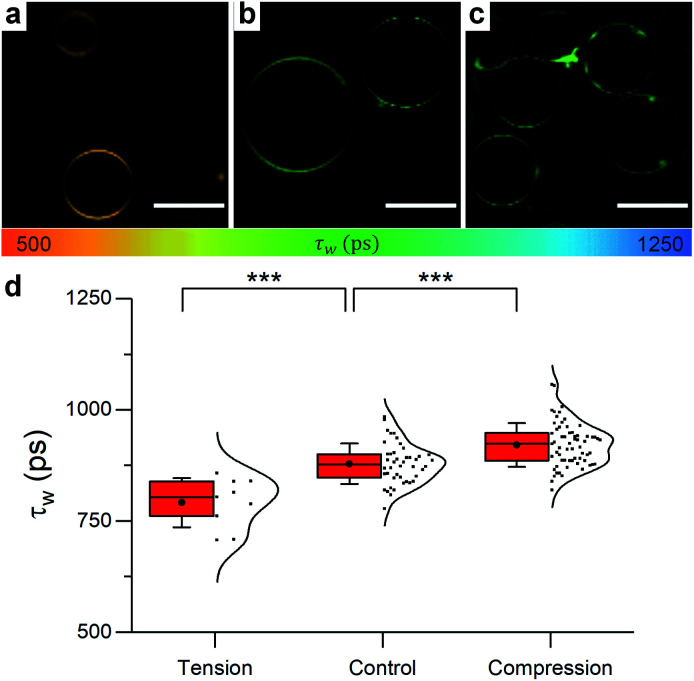
Example FLIM images of DOPC GUVs under (a) hypo-osmotic (Δ*C* = 0.18 M) (b) iso-osmotic (Δ*C* = 0 M) and (c) hyper-osmotic (Δ*C* = −0.36 M) conditions. (d) Average GUV *τ*_w_*n* ≥ 10. Scale bar: 30 μm.

Initially, pure DOPC GUVs were used to validate the previous results. Under zero tension, lifetime of 1 was slightly higher for DOPC GUVs than for LUVs of identical composition (Fig. 5), possibly due to DOPC oxidation during the electroformation process or smaller number of bilayer defects in GUVs compared to LUVs,^81^ which can increase membrane microviscosity.^82^

When osmotic stress was applied to these single-phase vesicles, the observed behaviour was identical to that in LUVs, *i.e.* DOPC GUVs microviscosity decreased with increased tension. In addition, stretching the membrane porated some of the vesicles, while compressing it led to excess area that was sometimes relieved by ejection of smaller lipid structures (Fig. S9†). Furthermore, the change in the lifetime of 1 was more pronounced when the bilayer was stretched, consistent with the existing theory (Fig. S15†).

**Fig. 6:**
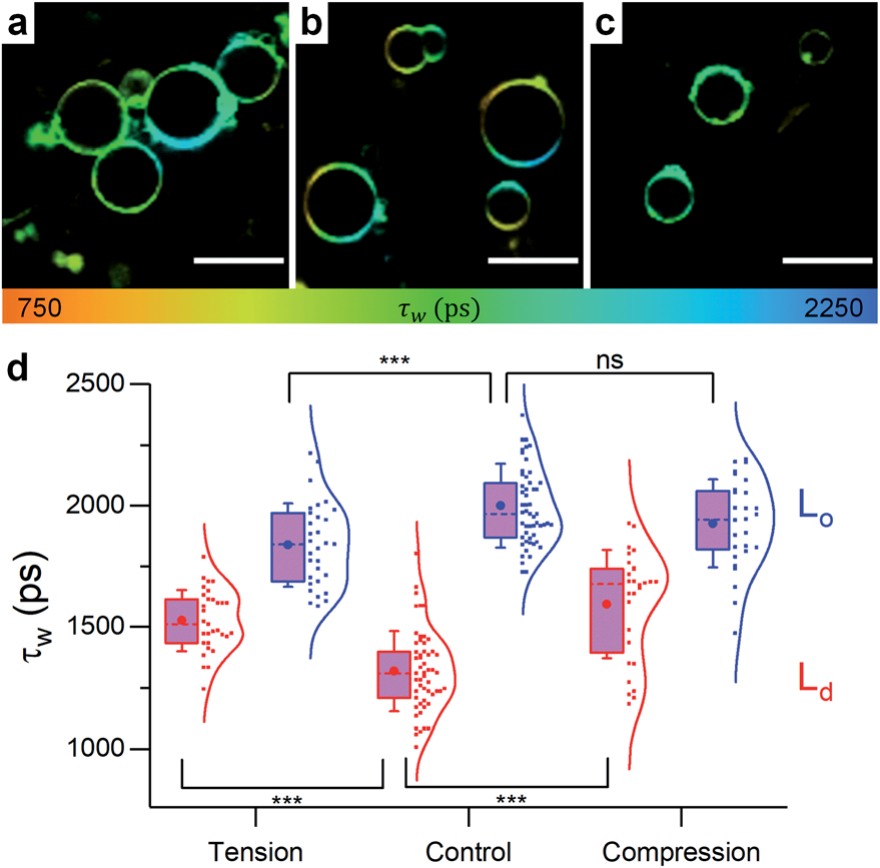
Example FLIM images of 40 : 40 : 20 DOPC : DPPC : cholesterol GUVs under (a) hypo-osmotic (Δ*C* = 0.18 M) (b) iso-osmotic (Δ*C* = 0 M) and (c) hyper-osmotic (Δ*C* = −0.36 M) conditions. L_o_/L_d_ coexistence is evident, *τ*_w_ for L_d_ and L_o_ phases are shown in (d). *n* ≥ 10. Scale bar: 30 μm.

A real advantage of our approach is being able to directly monitor phase separation and the corresponding microviscosity changes in both phases, with diffraction-limited resolution. In 40 : 40 : 20 DOPC : DPPC : cholesterol GUVs L_o_/L_d_ phase coexistence was detected, and the lifetime of 1 in the L_d_ phase was higher than that of pure DOPC as shown in Fig. 6. This observation is consistent with cholesterol partitioning in the L_d_ phase,^70^ and in agreement with the observed lifetimes of 1 in DOPC : cholesterol LUVs (Fig. S5†).

In contrast to pure DOPC GUVs, when stretched, pore formation was not frequently observed in phase separated GUVs (Fig. S9†) and the overall microviscosity of phase-separated GUVs remained unchanged, Fig. S10,† in agreement with the LUV data. However, the microviscosity of the L_d_ phase increased while that of the L_o_ decreased, yet separated domains could still be visualised (Fig. 6). The increase of viscosity of the L_d_ phase upon stretching is an unexpected result and it was not observed in any of the DOPC : cholesterol LUV compositions tested, indicating that the presence of saturated lipid is crucial for this behaviour. This result was further confirmed by studying ternary lipid compositions where DPPC was substituted with EYSM, where, again, an increase in L_d_ phase viscosity upon tension was observed (Fig. S11†).

The increase in membrane rigidity and microviscosity, assuming both are determined by lipid packing density, with increasing tension is known as the *strain-hardening* effect, which has been reported to take place in red blood cells (RBCs) and endothelial cells under tension (from hypo-osmotic shock^83^ or high shear^84,85^), as well as in malaria-infected RBCs.^86^ This behaviour has been usually attributed to a rearrangement of cytoskeletal fibres: either actin^87^ or intermediate filaments (keratin).^84,88^

To our knowledge, however, our work is the first instance were the *strain-hardening* effect has been mapped in model bilayers, in the absence of cytoskeleton. As suggested by our data, the *strain-hardening* effect is mediated by lipid diffusion from L_o_ domains into the L_d_ matrix. This is supported by both the higher frequency of domain merging seen in GUVs (*i.e.* the number of vesicles in which no phase separation could be identified, Fig. S12†) and the FRET domain mixing assay performed on LUVs (Fig. S13†). Both assays further confirm the tension buffering *via* L_o_-mediated diffusion hypothesis and agree with existing work where lipid domains were suggested to act as lipid reservoirs, key for stress buffering in Langmuir monolayers.^89–91^ These observations could be important in biology, aside from the effect on membrane mechanics, since the disruption of more ordered domains under tension was proven to trigger mechanosensitive signalling cascades in eukaryotic cells.^92,93^

On the other hand, membrane compression resulted in the increase of L_d_ viscosity while no change was seen for the L_o_ phase (Fig. 6). The net result in GUVs, therefore, is a slight increase of the average lifetime of 1, compared to the decrease observed in LUVs. Based on Fig. S9 and S12,† it appears that GUVs with a 40 : 40 : 20 DOPC : DPPC : chol composition react to osmotic pressure by ejecting lipid material as a mechanism for stress relieve, as was previously suggested in a number of studies.^94–96^

We hypothesize that budding off and membrane fission are the dominant mechanisms for relieving compression stress in GUVs. This process may be less likely in the case of significantly smaller LUVs. Additionally, lipid buds that were formed in LUVs will still be detected by a bulk spectroscopic measurement and therefore the pressure-induced softening may not be observed. The transition from the bilayer's buckling to budding and fission would be further facilitated by the higher compressive forces experienced by GUVs (Fig. S15†).

Further increasing cholesterol concentration to 50% (25 : 25 : 50 DOPC : DPPC : Chol GUVs) resulted in a single L_o_ phase (Fig. S14†). In this case, however, the average GUV viscosity increased under both tensile and compressive stress; contrary to what was seen in experiments using LUVs (Fig. 2). The increase in ordering under tension can be explained by the emergence of phase-separated domains,^32^ less likely to form in more curved membranes (as highly ordered domain formation is favoured in planar geometries^21,22^). Above Δ*C* = 0.18 M, the microviscosity of the new L_o_ phase is similar to the one measured in the 40 : 40 : 20 GUVs, however the lifetime of 1 in the L_d_ phase is significantly increased. This could be due to the increased fraction of cholesterol partitioning in the DOPC rich phase.^70^ The increase in viscosity under compression could be compared to that in 40 : 40 : 20 GUVs. As stress is released by fission of membrane buds, the curvature-induced softening of the more ordered L_o_ phase is not triggered and, instead, an increase of the overall viscosity due to compression of the unsaturated lipid chains is observed.

It must be noted though that results obtained by FLIM imaging should be interpreted with care. The spatial resolution is limited (particularly due to pixel binning required to attain a level of signal necessary for lifetime fitting), which can prevent the detection of smaller domains.

**Fig. 7:**
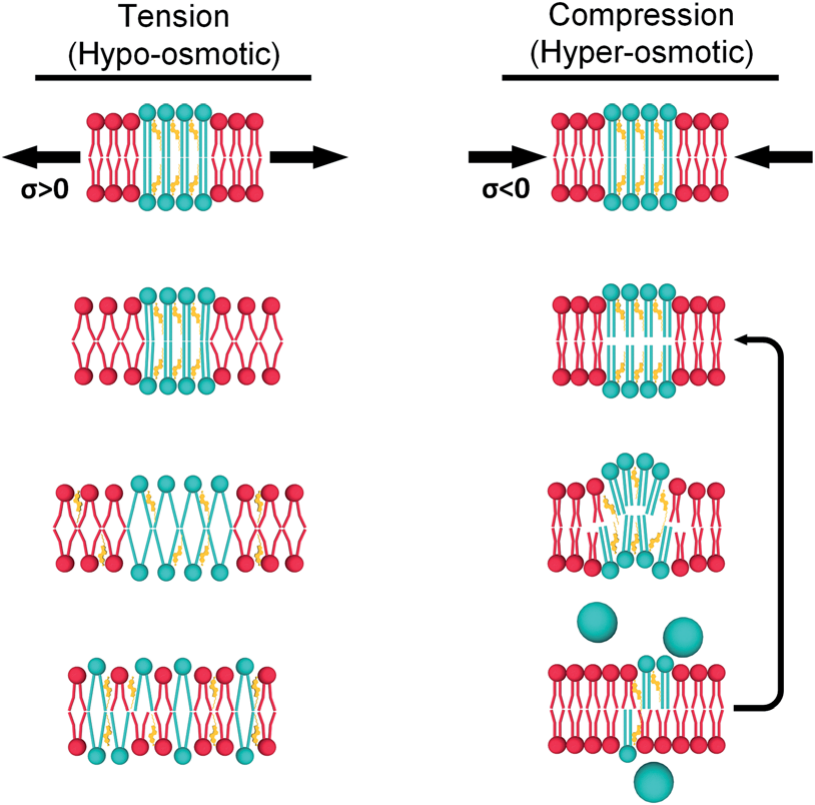
Hypothetical mechanisms for tension (left) and compression (right) buffering. When tension is applied, the L_d_ phase (red) expands and tension is ultimately transmitted to the L_o_ domains (green). Enough tension would decrease lipid packing in the L_o_ phase, but this is unfavourable and instead the L_o_ phase solubilizes into the L_d_ matrix. In contrast, increased compression would initially increase packing of DOPC molecules in the L_d_ phase. This would be followed by buckling out of the L_o_ domains and, eventually, ejection of excess lipid to relieve membrane stress (the last step is more likely to happen in larger GUVs).

Based on all our results, in LUVs and in GUVs, we propose two mechanisms by which lipid bilayers can minimize mechanical stress (Fig. 7):

(1) Under load, unsaturated lipids with lower area expansion modulus (*K*_A_) such as DOPC,^97^ will be the first to deform. This is reflected by a change in viscosity of the L_d_ phase.

(2) The membrane stress will be ultimately transferred to the L_d_/L_o_ interface, and the more ordered domains will respond differently for tension and compression forces.

#### Tension

Increased tension will cause merging of L_o_ domains in order to minimize the edge energy at the phase interface.^35^ Upon reaching a critical tension, L_o_ domains will become unstable^27,31,33^ and DPPC will solubilize in the L_d_ matrix, filling the void between DOPC molecules. This will buffer the fluidity increase expected for pure DOPC membranes.

#### Compression

However, if the lipid bilayer is compressed, the L_o_ phase will ripple and buckle, locally softening and increasing its disorder (negative compressibility). Ultimately, excess lipid could also be ejected to help relieving membrane stress, which in turn could prevent membrane softening. The latter is more likely to happen in GUVs compared to smaller LUVs.

## Conclusions

By using viscosity sensitive probes, molecular rotors, that effectively incorporate in all lipid phases formed in DOPC : DPPC : cholesterol vesicles of varied compositions, our results suggest that lipid membranes behave as complex materials, which show non-classical mechanical behaviour. Particularly, bilayers showing L_o_/L_d_ phase separation can buffer tensile efforts, while gel phases show negative compressibility under pressure. The former, considered a prototype for lipid rafts, emphasises their possible role in maintaining the cell's adaptability to external stress.

## Materials and methods

Lipids 1,2-dioleoyl-*sn-glycero*-3-phosphocholine (DOPC), 1,2-dipalmitoyl-*sn-glycero*-3-phosphocholine (DPPC), 1-palmitoyl-2-oleoyl-*sn-glycero*-3-phosphocholine (POPC), 1,2-diphytanoyl-*sn-glycero*-3-phosphocholine (DPhPC), egg yolk sphingomyelin (EYSM) and biotinylated 1,2-dipalmitoyl-*sn-glycero*-3-phosphoethanolamine (Biotin@PE) were purchased in powder form from Avanti Polar Lipids® and resuspended in chloroform (20 mM) before use. Fluorescent lipids 1,2-dipalmitoyl-*sn-glycero*-3-phosphoethanolamine-*N*-(lissamine rhodamine B sulfonyl) (ammonium salt) (Rh@PE) and 1,2-dioleoyl-*sn-glycero*-3-phosphoethanolamine-*N*-(7-nitro-2-1,3-benzoxadiazol-4-yl) (ammonium salt) (NBD@PE) were obtained as chloroform solutions (1 mg mL^−1^) from Avanti Polar Lipids®. The thiophene-based dyes 1 and 2 were synthesised according to the previously published procedure.^62^ Stock solutions of 1 were prepared in DMSO (3 mM) or in CHCl_3_ (300 μM). All other reagents were purchased from Sigma Aldrich® or VWR and used without further purification. Solvents for fluorescence studies were of spectrophotometric grade.

### Rotor calibration

Mixtures of 0, 40, 90 and 100% (v/v) of castor oil in toluene were prepared, and 1 was dissolved to a final concentration of 6 μM by heating each solution above 70 °C. Time resolved fluorescence decays were measured from each of these solutions as described below at a range of temperatures 20–60 °C, providing a viscosity range of 2–1000 cP.^52^

### Large unilamellar vesicle (LUV) formation

A dried lipid film was created by mixing the lipid stock solutions at the appropriate DOPC : DPPC : cholesterol molar ratio. An aliquot of 1 or BODIPY molecular rotor (both 1 mM in CHCl_3_) were added at a 1 : 400 dye : lipid ratio, before using a rotary evaporator to remove the solvent and to create a lipid film. Deionised water or 0.4 M aqueous sucrose solution, for osmotic pressure experiments, were then added to hydrate the film to a final concentration of 1 mM lipid. The mixture was then vortexed and extruded 21 times through a 200 nm (unless otherwise stated) polycarbonate filter (Avanti Polar Lipids®).

Alternatively, pure lipid vesicles were prepared as described above and an aliquot of 1 in DMSO (1 mM stock solution) was externally added to achieve 1 : 400 dye : lipid ratio. The final concentration of DMSO was 0.5% v/v. The mixtures were incubated for at least 15 min above the melting temperature of the lipid (*T*_m_). This was done to better mimic experiments involving cells, where the rotor is added to the plasma membrane from the aqueous phase. At these conditions, an excellent linear cross-calibration of the fitted lifetime of 1 with that of the well characterized BODIPY rotor^56^ (Fig. S16†) is seen, confirming its usefulness as a molecular rotor.

LUVs were then diluted by a factor of 10 (using aqueous solutions of different glucose concentration for osmotic pressure experiments) before the measurement. Dynamic Light Scattering (Malvern Panalytical, Zetasizer Ultra) was used to confirm vesicle size to be within the expected range (∼180 nm for 200 nm extrusion filter, data not shown).

### Giant unilamellar vesicle (GUV) formation

30 μL of a 1 mg mL^−1^ lipid solution (of the appropriate DOPC : DPPC : cholesterol ratios (DPPC was substituted for EYSM when indicated), supplemented with 1% Biotin@PE and either 0.5% Rh@PE or 1% of 1 in CHCl_3_ at 1 mM) was spread onto a heated ITO slide. After drying for >1 h in a desiccator, a chamber was created by placing a polydimethyl siloxane (PDMS) spacer (∼2 mm thick) on top, which was then filled with 0.4 M sucrose solution and sealed with a second ITO slide. An electric field of 1.5 V_pp_@10 Hz was applied for 90 min, followed by a detachment phase of 1.5 V_pp_@2 Hz for 30 min. GUVs were diluted ten-fold before imaging.

### Osmotic pressure experiments

Vesicles containing 0.4 M sucrose were diluted ten-fold in solutions with different glucose concentration, creating an osmotic gradient. Measurements were carried out after at least 5 min equilibration time. For the FRET experiments, the lipid mixture in CHCl_3_ was doped with 0.2%mol NBD-PE and 2%mol Rh-PE before creating the lipid film.

### Fluorescence spectra and lifetime measurements

Samples were placed in quartz cuvettes (10 mm path length). A Horiba Yvon Fluoromax 4 fluorimeter was used to record the emission spectra under 467 nm (for pure 1) or 460 nm (for FRET experiments) excitation, which were corrected for the wavelength-dependent emission detection sensitivity. Time resolved fluorescence decay traces of 1 were acquired using a Horiba Jobin Yvon IBH 5000 F time-correlated single photon counting (TCSPC) instrument with detection at 570 ± 32 nm or 650 nm ± 8 nm, for LUVs and castor oil/toluene, respectively, after 467 nm pulsed excitation (NanoLED). In the case of BODIPY rotor, a 404 nm pulsed laser was used, and the emission was recorded at 515 ± 32 nm. Acquisition was stopped after peak counts reached 10 000, and the resulting traces were fitted to a biexponential decay using DAS® software. Temperature was controlled either with a Peltier cell (fluorimeter experiments, error: ±0.5 °C) or a water bath (TCSPC, error: ±1 °C) and samples were left to equilibrate for at least 5 min before each measurement.

### Widefield microscopy

Observation chambers were made with a PDMS well and glass was coated with BSA@biotin-streptavidin to immobilize the GUVs. A Nickon Eclipse TE2000-E inverted microscope was used to acquire phase contrast and fluorescence images. Excitation was provided by a mercury arc lamp together with suitable filters.

### Fluorescence lifetime imaging microscopy (FLIM)

A Leica TSC SP5 II inverted confocal microscope with a 63× water immersion objective (NA: 1.2) was used to acquire lifetime images. Two-photon excitation at 900 nm was provided by a Ti:sapphire laser (Coherent, Chameleon Vission II 80 MHz), and fluorescence emission was collected between 500–700 nm. FLIM images were recorded using a TCPSC card (Becker and Hickl GmbH, SPC-830) with a size of 256 × 256 pixels and 256 channels. The IRF was obtained by measuring second harmonic generation (SHG) signal from urea. SPCImage (Becker & Hickl GmbH®) was used to fit the decays, ensuring a minimum of 200 counts per pixel (after binning). Sample preparation was the same as with wide-field microscopy.

### Lifetime classification into lipid phases

A custom-built MATLAB® script was used to identify phase separation. Briefly, GUVs were manually selected, and a lifetime histogram was created from the resulting ROI. Lifetimes were then divided into 3 groups using K-means classification (ensuring a threshold difference between them) and the groups were manually assigned to either L_d_/L_o_ phase or to a single phase. The middle cluster was assigned either to one of the phases or remained separated (in which case it was considered as an intermediate viscosity state between the L_d_ and L_o_ regions).

### Sample preparation for X-ray measurements

A dry sample of a given lipid mixture (20 mg final lipid mass) was hydrated with excess water (70%) and subjected to ten freeze–thaw cycles to ensure proper lipid mixing. The sample was then loaded into 2 mm diameter polymer capillary tube, sealed with a rubber stopper, and stored at 4 °C until further use.

### High pressure X-ray diffraction

SAXS and WAXS measurements were carried out at beamline I22 at Diamond Light Source (UK) using a custom-built high-pressure cell. Radial integration of 2D SAXS and WAXS patterns gave the scattering intensity profiles. Peak intensity and position were obtained after baseline subtraction and fitting to one or two Voigt functions using a custom-built MATLAB® script. See ESI† for more details.

### Statistical analysis

Data is shown as mean ± S.D. Box plots display the 25–75% range, error bars represent ±S.D., median is shown by a horizontal line and mean by a dot. Origin® software was used to perform one-way ANOVA test. *p < 0.05; **p < 0.01; ***p < 0.001.

## Conflicts of interest

There are no conflicts to declare.

## Supplementary Material

SC-012-D0SC05874B-s001
