# Supplementary material for: Imaging non-classical mechanical responses of lipid membranes using molecular rotors
Source: Chem Sci. 2020 Dec 22;12(7):2604–13. doi: 10.1039/d0sc05874b (PMC8179291; doi:10.1039/d0sc05874b)
Supplement: SC-012-D0SC05874B-s001 [file SC-012-D0SC05874B-s001.pdf]

Electronic Supplementary Material (ESI) for Chemical Science.
This journal is © The Royal Society of Chemistry 2020

Supplementary Information:

Imaging non classical mechanical response of lipid membranes using molecular rotors

Miguel Páez-Pérez[a], Ismael Lopez-Duarte,[b] Aurimas Vyšniauskas[c], Nicholas J. Brooks[a*] and Marina K. Kuimova[a*]

[a] *MSRH, Department of Chemistry, Imperial College London, Wood Lane, London, W12 0BZ, UK*
[b] *Departamento de Química Orgánica, Universidad Autónoma de Madrid, Cantoblanco, 28049 Madrid, Spain*
[c] *Center of Physical Sciences and Technology, Saulėtekio av. 3, Vilnius, Lithuania*

Supplementary data:

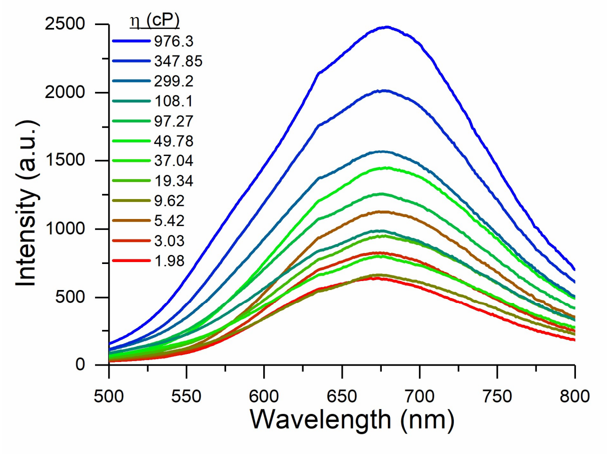

Fig. S1: Emission spectra of **1** recorded in castor oil/toluene calibration mixtures, which appear to be composed of more than one Gaussian band. Peak wavelength did not change significantly at different castor oil ratios.

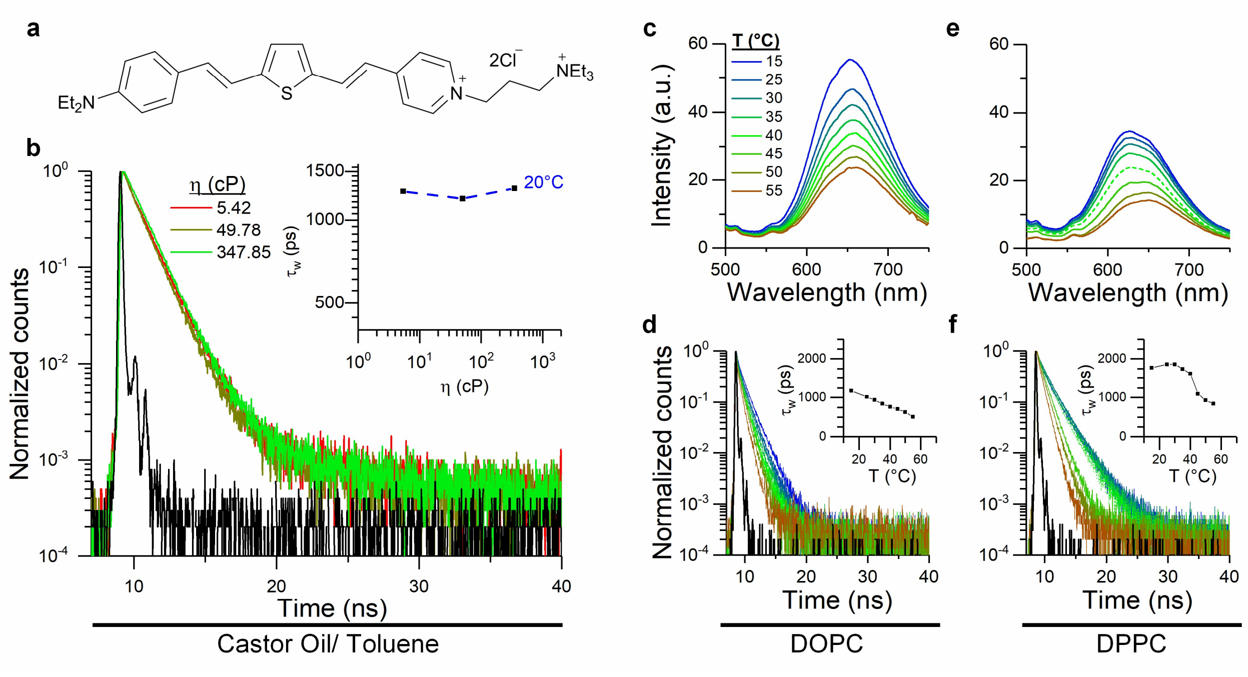

Fig. S2: Characterization of the viscosity response of dye **2.** (a) Molecular structure of **2**; (b) Time resolved fluorescence decay traces recorded for **2** at 20°C and different castor oil/toluene ratios. Decay traces and calculated τ_w (insert) show no dependence on viscosity; (c,e) emission spectra and (d,f) decay traces and calculated τ_w of **2** recorded in DOPC and DPPC LUVs. The spectral changes observed for **2** upon the change in temperature when incorporated in lipid bilayers occur due to altered polarity and/or hydration of the membrane, similar to Laurdan-type dyes.

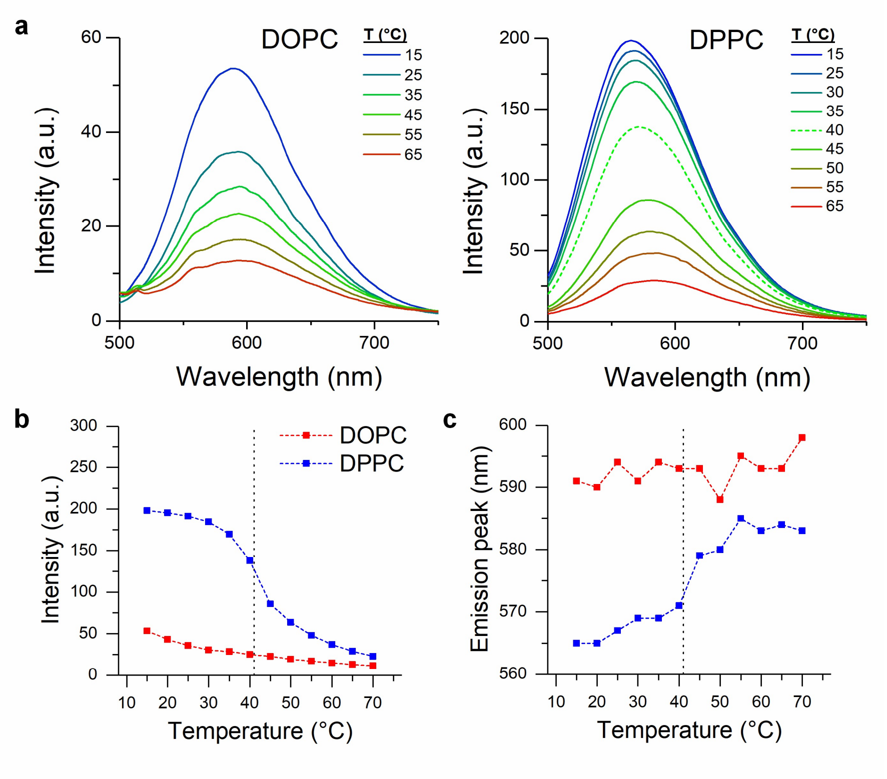

Fig. S3: Spectral response of **1** recorded in DOPC and DPPC LUVs at a range of temperatures. (a) Fluorescence emission spectra; (b) emission peak intensity; (c) emission peak wavelength. The vertical dotted line represents T_m expected for DPPC (41°C).

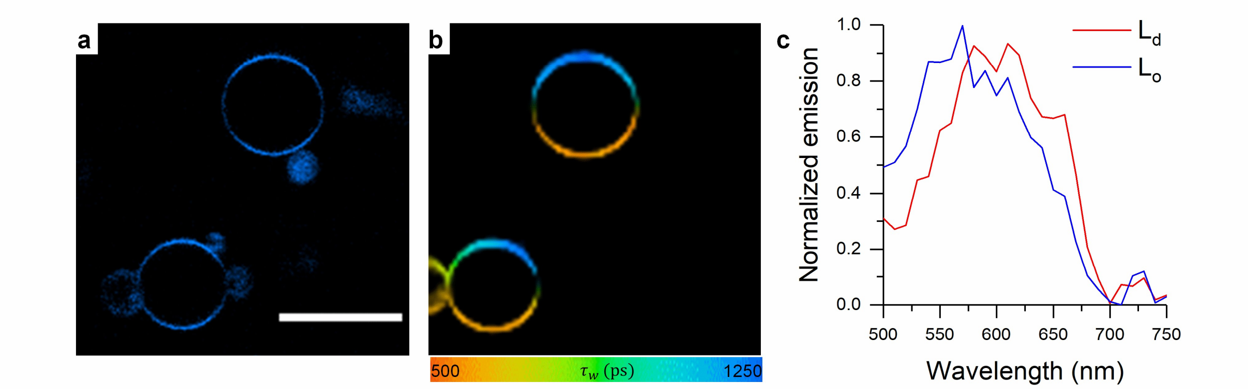

Fig. S4: 1 localization and fluorescence in 40:40:20 GUVs. (a) Emission collected between 500 and 700nm; (b) FLIM image of the GUVs and (c) emission spectra (averaged from both GUVs) of the L_d and L_o phases. The higher hydration of the fluid phase is reflected as a red-shift of the spectra. Scale bar: 30µm.

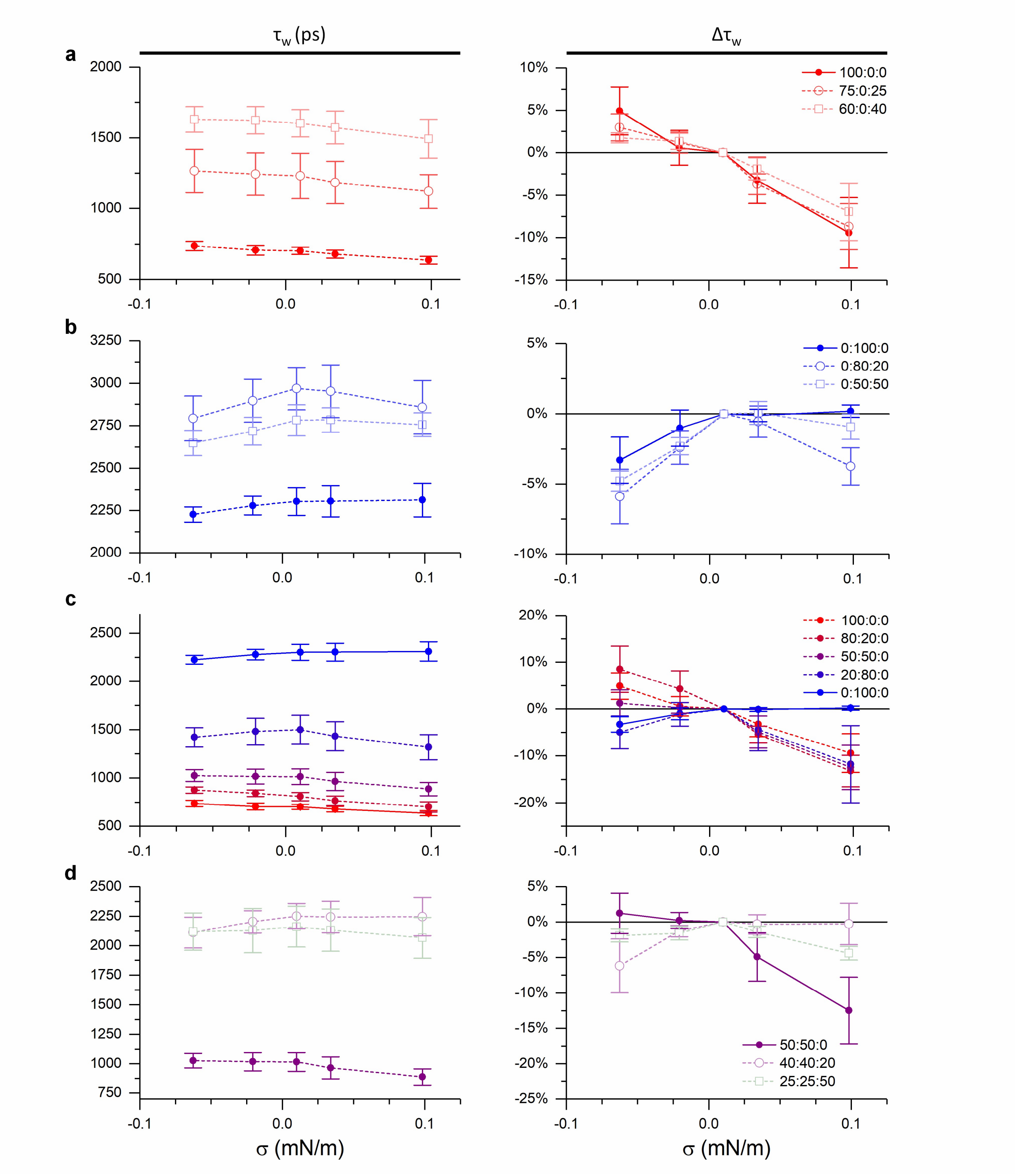

Fig. S5: 1 lifetime (absolute value and relative change compared to iso-osmotic conditions) for 200nm diameter LUVs of different compositions under stress. (a) binary DOPC/Cholesterol mixtures; (b) binary DPPC/Cholesterol mixtures; (c) binary DOPC/DPPC mixtures and (d) ternary DOPC/DPPC/Cholesterol mixtures.

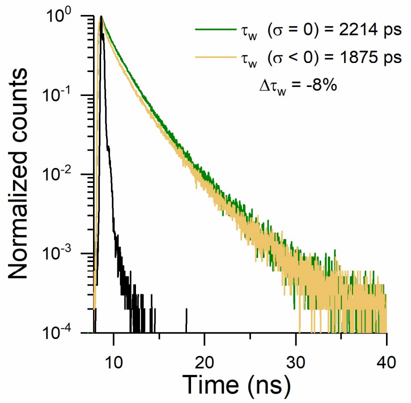

Fig. S6: Further evidence of negative compressibility of lipid bilayers in gel phase. The well-characterized BODIPY[4] rotor was added externally (in the aqueous phase) and, under osmotic pressure, reflected a decrease in membrane viscosity.

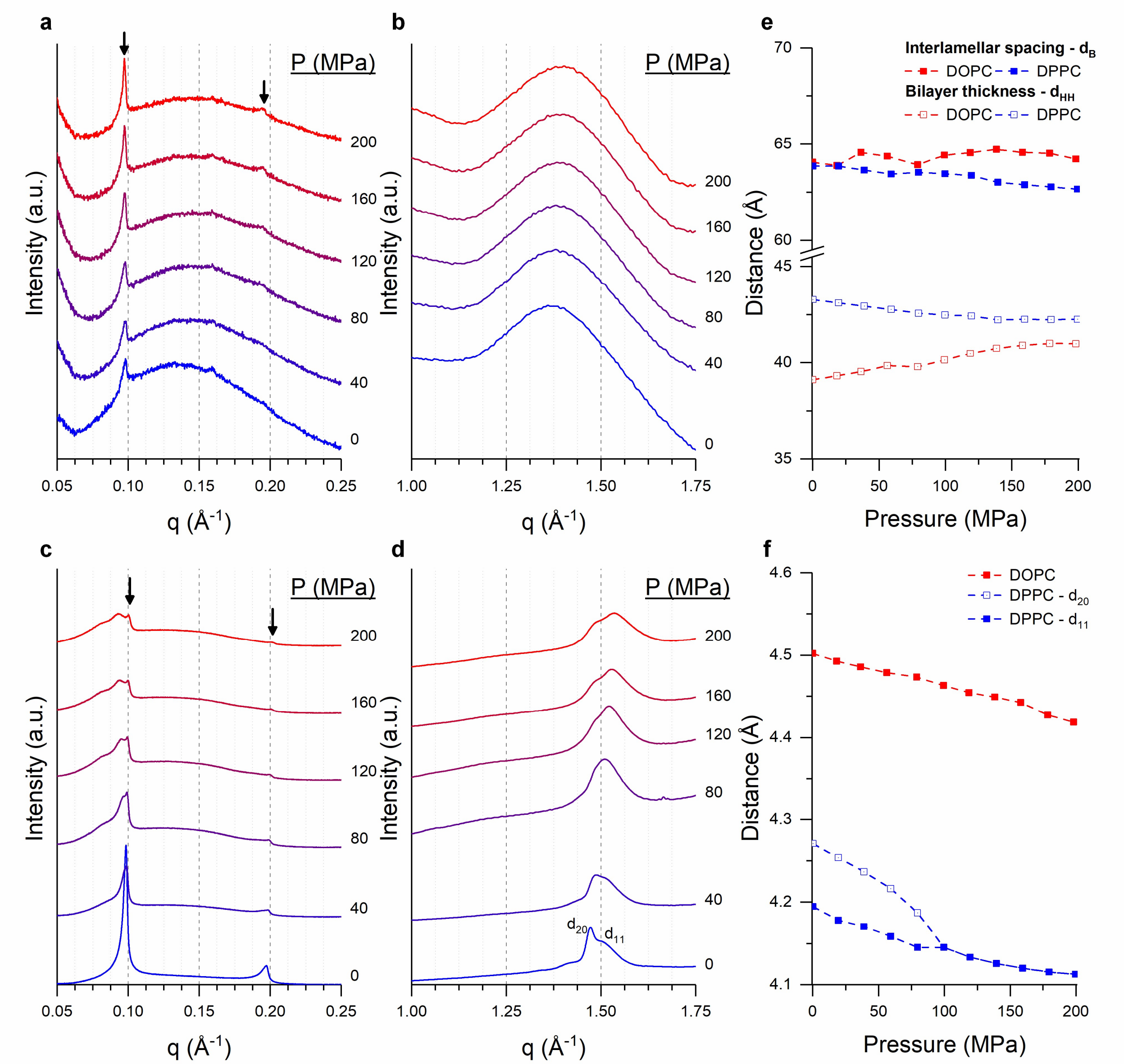

Fig. S7: X-ray scattering intensity profiles for DOPC and DPPC at 70% hydration. (a,b) SAXS and WAXS profiles of DOPC at increasing pressure. (c,d) SAXS and WAXS profiles of DPPC at increasing pressure. (e) Interlamellar spacing (calculated from SAXS 1st order peak position, as $d=2\pi/q$) and bilayer thickness estimation using the first two peaks, following the procedure indicated by *Rappolt et. al*[5]. (f) Inter-chain spacing calculated from WAXS peak position. For DPPC at low pressures, two peaks were fitted.

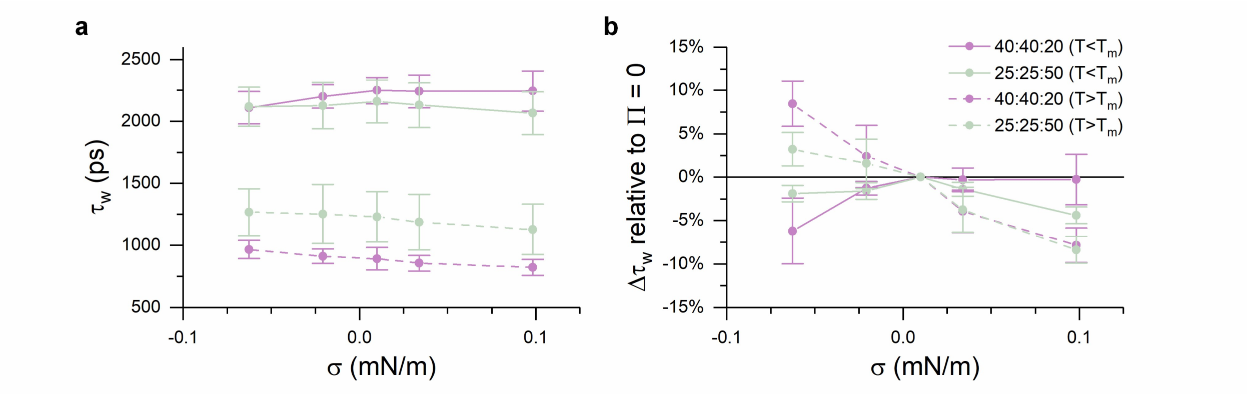

Fig. S8: Effect of temperature on stress buffering by LUVs. (a) intensity-weighted average lifetime of **1**. (b) Change in the lifetime of **1** relative to zero tension.

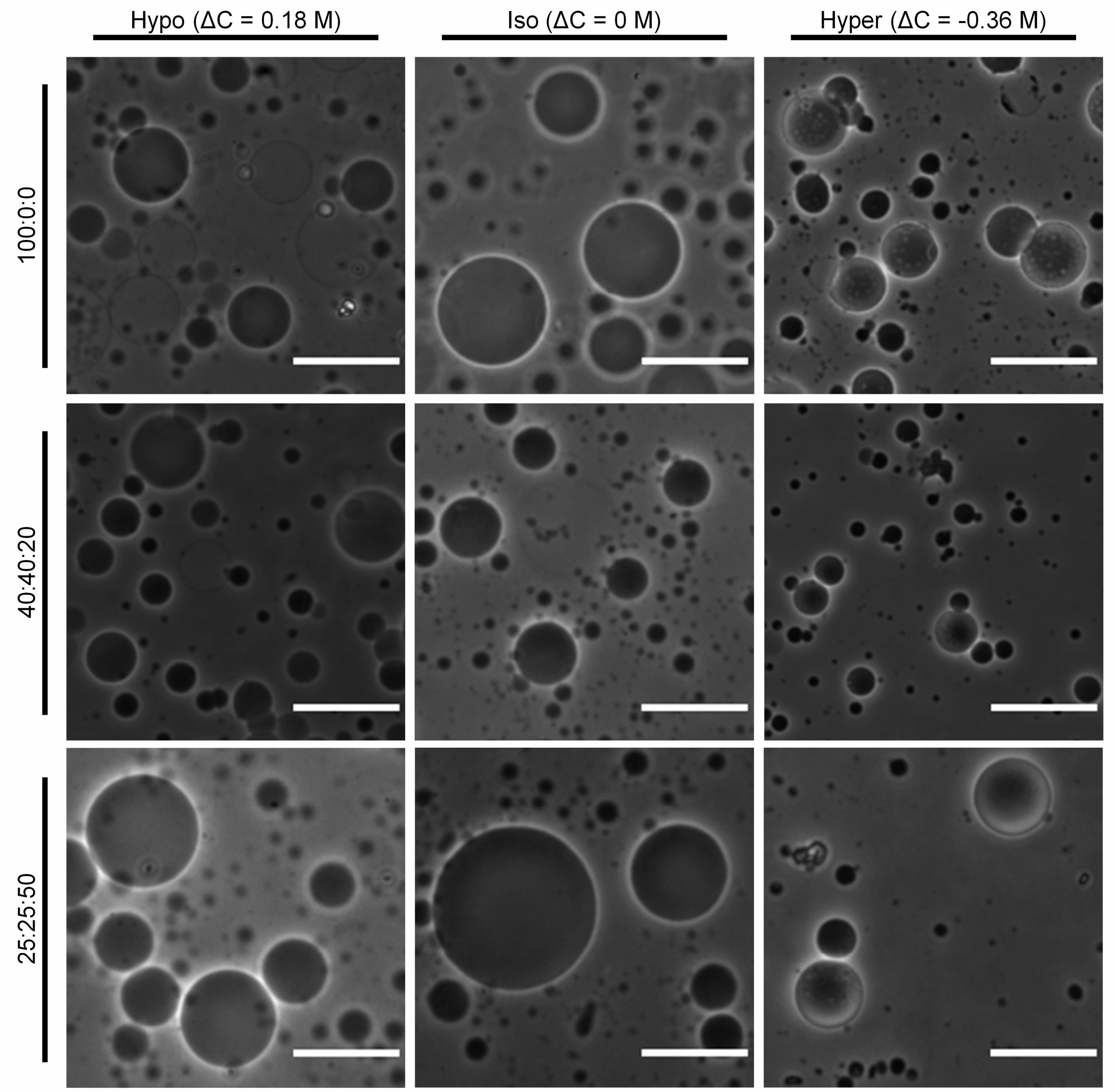

Fig. S9: Phase contrast images of the GUVs of different composition under the conditions described in the main text. The presence of contrast between the GUV and its surroundings was linked to the lack of pore formation. Scale bar: 30μm.

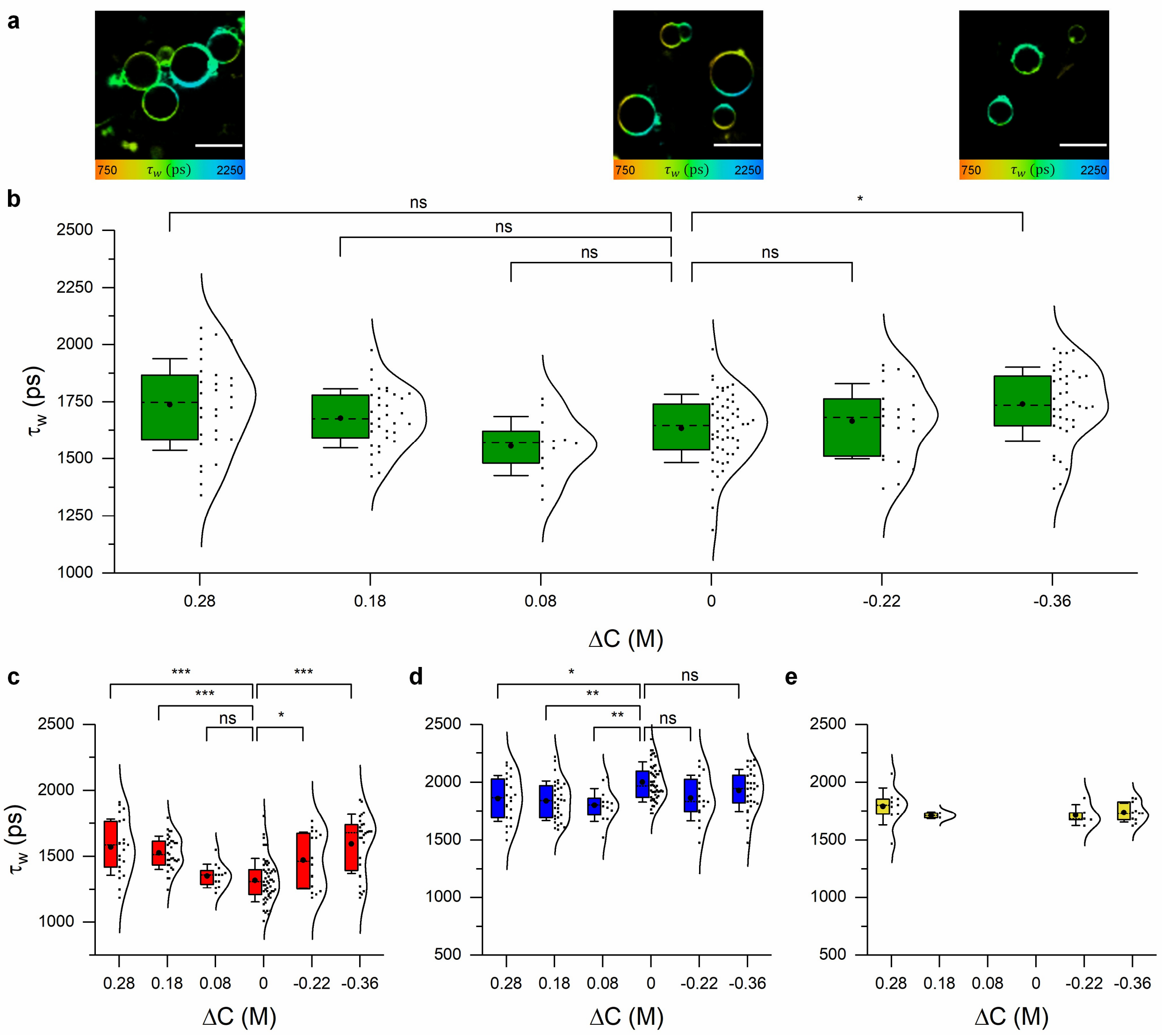

Fig. S10: The fluorescence lifetime analysis of **1** incorporated into 40:40:20 GUVs under different osmotic conditions. (a) FLIM images at the conditions shown in the main text; (b) Average GUV lifetime; (c) L_d; (d) L_o and (e) lifetime when no two phases could be distinguished (i.e. single phase GUV). Scale bar: 30μm

Increased tension leads to L_o disassembly (hence the viscosity recovery) and hardening of the L_d phase. At this points some single-phase GUVs can be seen. On the contrary, membrane compression increases the viscosity of the L_d phase.

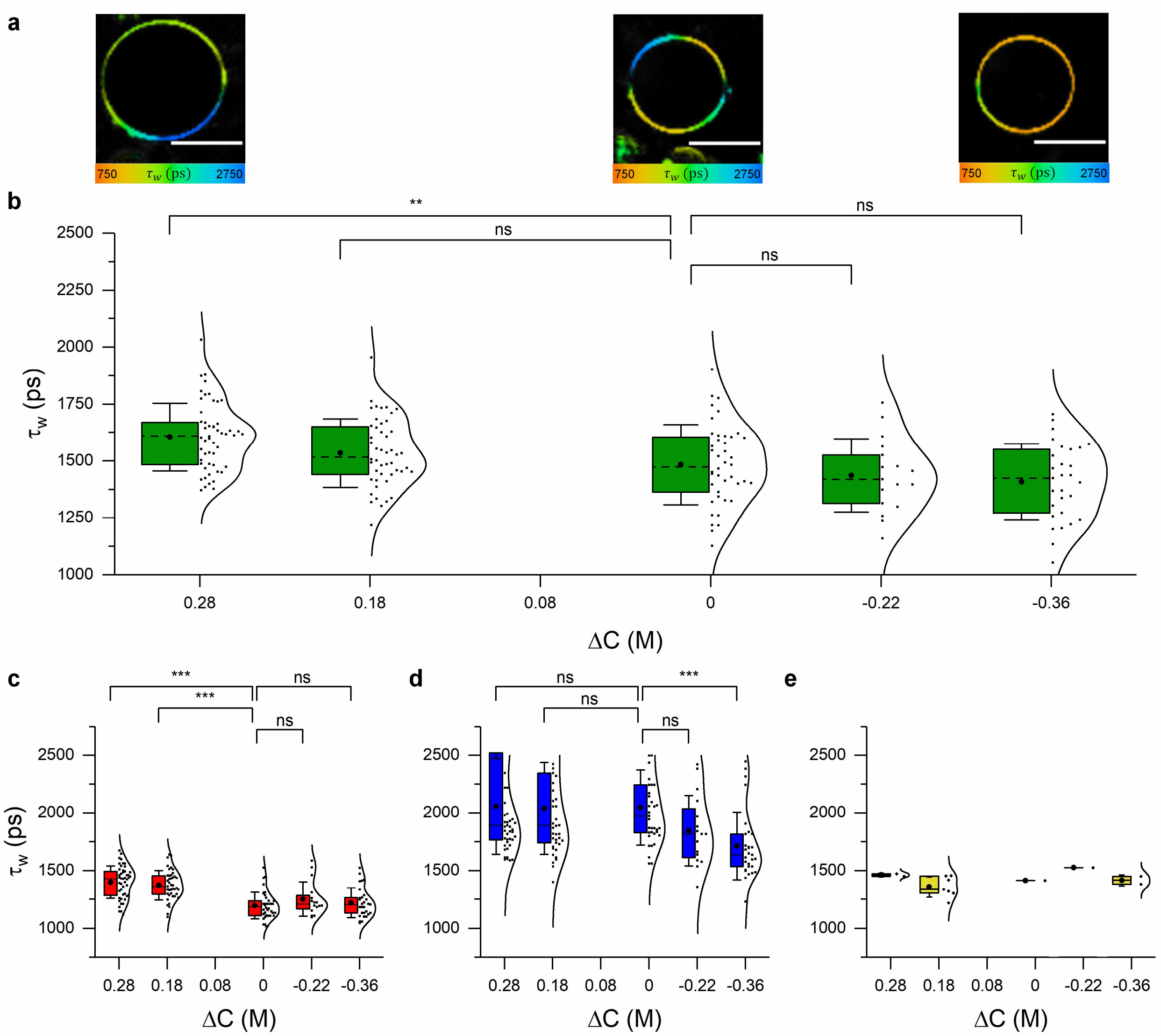

Fig S11: The fluorescence lifetime analysis of **1** incorporated into 40:40(EYSM):20 GUVs under different osmotic gradients. (a) FLIM images; (b) Average GUV lifetime; (c) L_d; (d) L_o and (e) lifetime when no two phases could be distinguished (i.e. single phase GUV). Scale bar: 30µm

Like GUVs having DPPC as the saturated lipids, viscosity of L_d domains in EYSM containing GUVs also increases upon tension. Interestingly though, a decrease in lifetime of the L_o phase can be identified, which was not observed in DPPC containing GUVs.

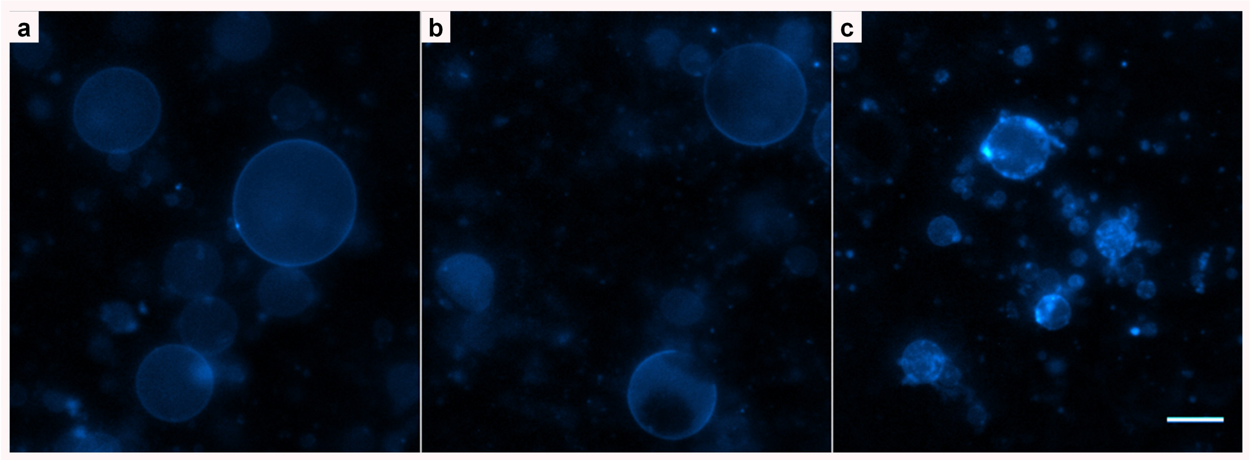

Fig S12: Rh@PE labelled 40/40/20 GUVs showing domain reorganization under osmotic pressure. Ld domains appear fluorescent. (a) Hypo-osmotic conditions - ΔC: 0.18 M (b) Iso-osmotic conditions - ΔC: 0 M (c) Hyper-osmotic media - ΔC: -0.36 M. Under tension, Lo domains disassemble resulting in homogeneous fluorescence while compression resulted in the ejection of lipid material. Scale bar: 20μm

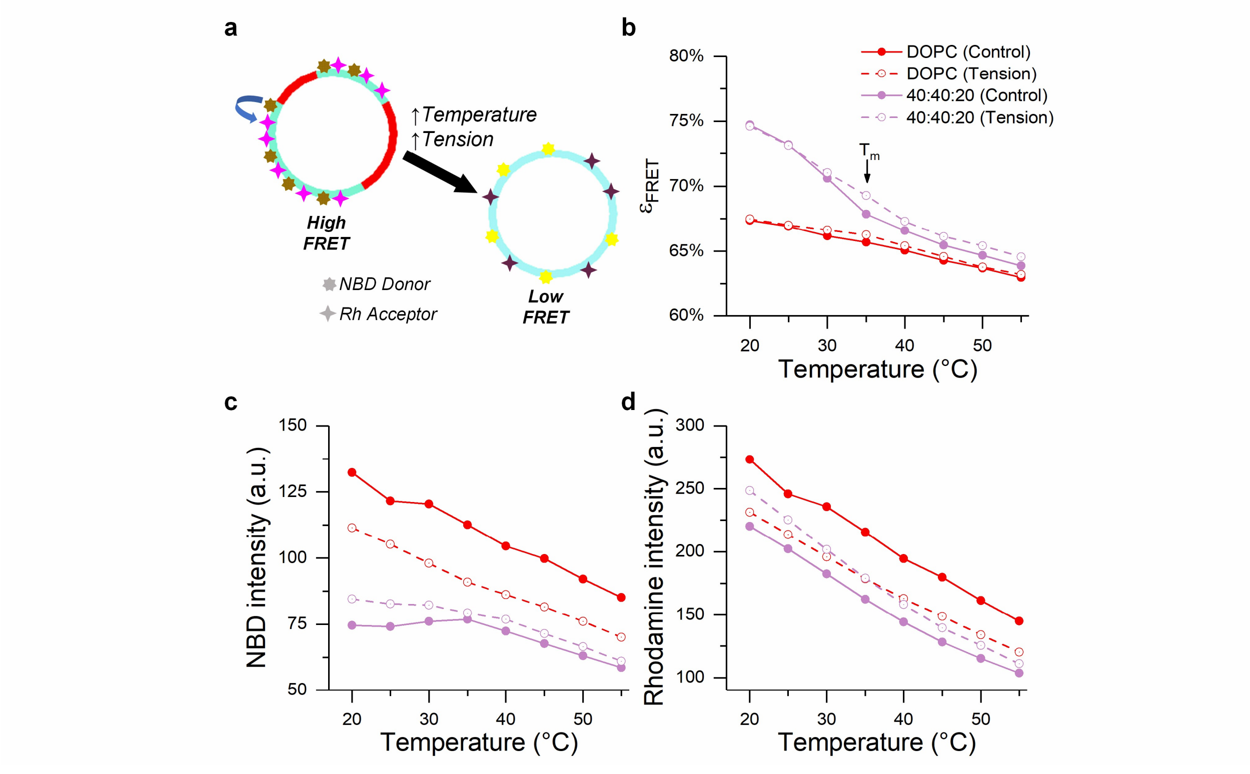

Fig. S13: NBD/Rhodamine FRET assay performed on LUVs suggests L_o domain diffusion into the L_d matrix. (a) When domains are present, NBD and Rhodamine labelled lipids are clustered together resulting in a higher FRET signal. However, when domains are no longer present, these lipids will be located further apart therefore FRET efficiency is reduced. (b) FRET efficiency calculated as A/D+A. No inflection point is seen for DOPC at any conditions, indicating no phase separation. A clear inflection point is seen for phase separated non-tensed 40:40:20 LUVs. However, 40:40:20 LUVs under tension do not display the inflection point, suggesting domains have disappeared, at least partially. (c) NBD emission. The existence of L_o domains is suggested by the slope change for the 40:40:20 composition under zero tension (control) at T_m = 35C. (d) Rhodamine emission. The steady decrease in fluorescence is attributed to the temperature sensitivity of this dye[6].

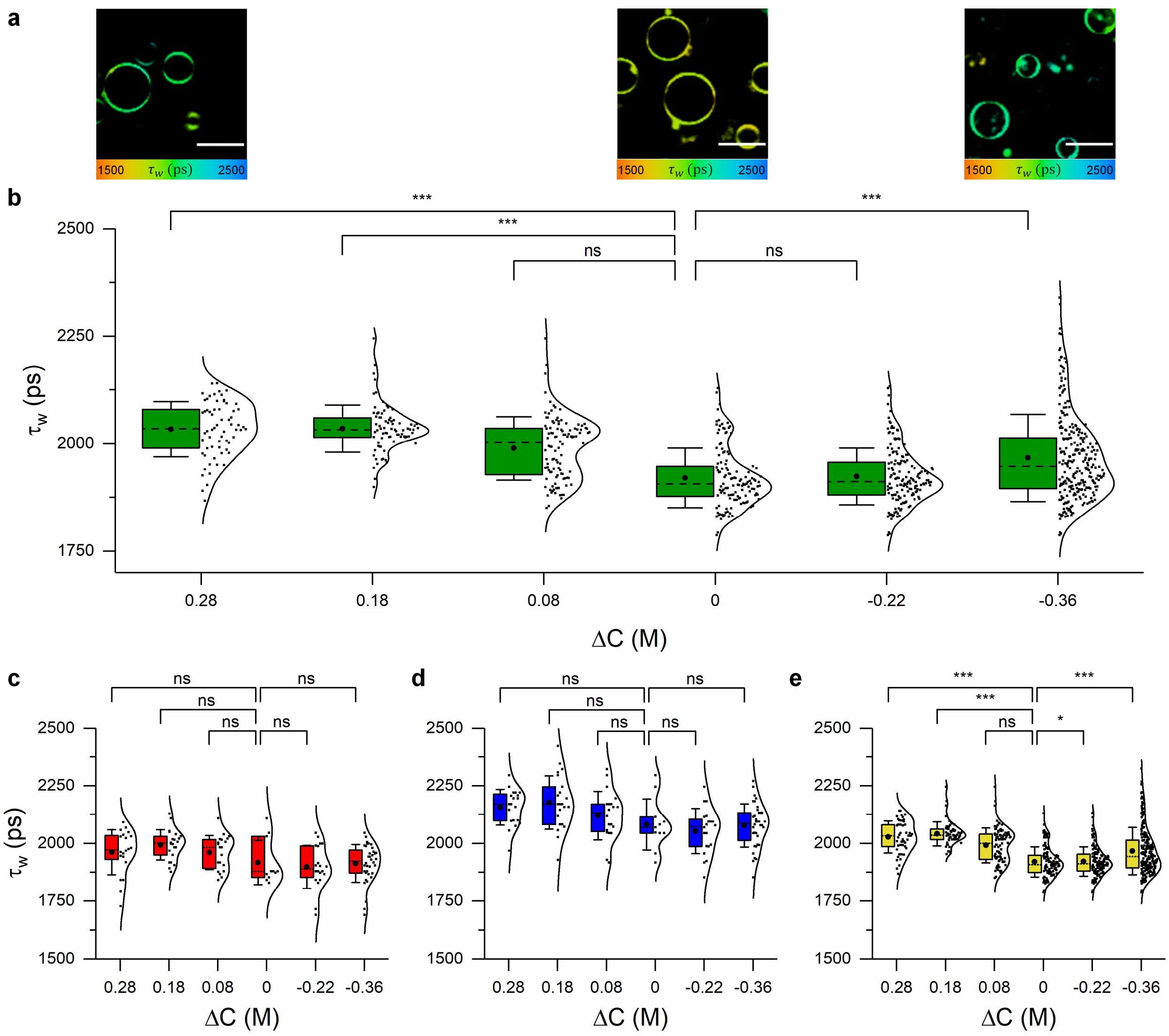

Fig. S14: The fluorescence lifetime analysis of **1** incorporated into 25:25:50 GUVs under different osmotic gradients for each of the phases. (a) FLIM images at selected conditions; (b) Average GUV lifetime; (c) L_d; (d) L_o and (e) lifetime when no two phases could be distinguished (i.e. single phase GUV). Scale bar: 30μm

Even a small tension increase (ΔC = 0.08) leads to an increase in overall viscosity, which we attribute to phase-separated domains which cannot be distinguished in the lifetime histogram. Larger tension leads to L_d/L_o domain coexistence. Membrane compression causes a linear-like increase in viscosity.

Discussion on membrane buckling:

The likelihood of membrane buckling increases with higher Föppl–von Kármán number (FvK), defined as:

$$FvK := 12(1-v^2)\left(\frac{R_0}{h}\right)^2$$

where v is the Poisson ratio, R_0 is the shell radius and h is the lipid bilayer's thickness[7]. Considering v = 0.5 and h = 4nm, FvK (R_0 = 100nm) = $5.625 \cdot 10^3$; hence the buckling hypothesis could be possible from a theoretical standpoint[8,9].

Calculation of membrane tension and viscosity response:

The tension across the bilayer (σ) can be expressed in terms of the osmotic pressure across the interface (P),the vesicle radius (r), bending modulus (κ) and spontaneous curvature (c_0)[10].

$$\sigma = \frac{Pr}{2} + \frac{\kappa c_0}{2r}(2 - c_0 r) \quad (1)$$

By considering $c_0 \approx 1/r$; and expressing P in terms of ΔC according to Van't Hoof's relation as P = RTΔC (where R is the ideal gas constant, T is the temperature and the concentration gradient, ΔC, is defined as ΔC:= C_{in}-C_{out}) we end up with:

$$\sigma = \frac{RT}{2} r\Delta C + \frac{\kappa}{r^2} \quad (2)$$

However, the vesicle's volume will change as water crosses the membrane to equalize the osmotic imbalance. Assuming the membrane is only water permeable, the final vesicle radius could be written as:

$$r = r_0 \sqrt[3]{C_{in}/C_{out}} \quad (3)$$

where r_0 is the initial vesicle size (at iso-osmotic conditions). Then (2) turns to be:

$$\sigma = \frac{RT}{2}\Delta C \left[r_0 \sqrt[3]{C_{in}/C_{out}} \right] + \kappa \left[r_0 \sqrt[3]{C_{in}/C_{out}} \right]^{-2} \quad (4)$$

Assuming, κ ~ ϑ(-19), r ≈ 100nm (for LUVs) and considering T = 298K, values for membrane tension at different concentration gradients can be calculated. As seen in Fig. S15, a higher tension would be experienced by larger vesicles.

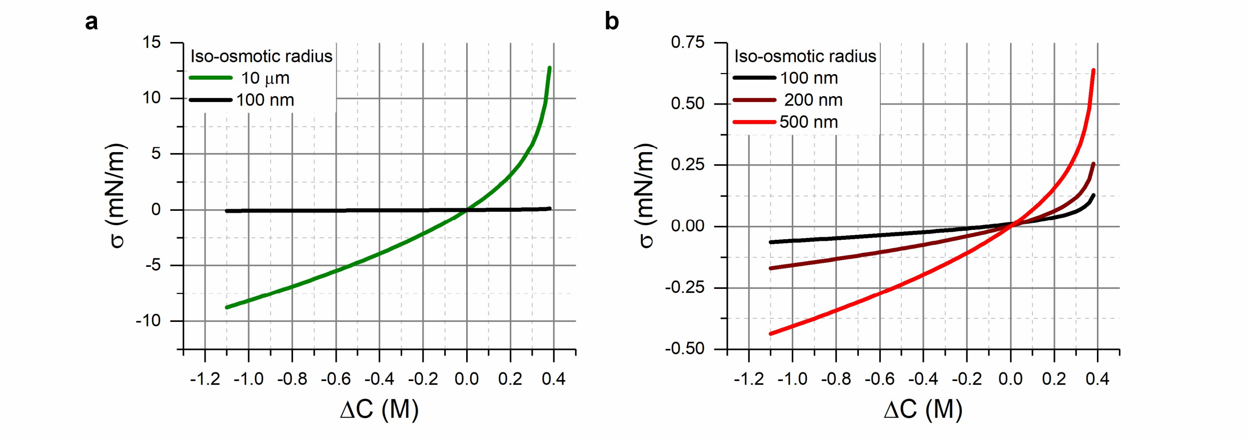

Fig. S15: Theoretical calculations of the membrane tension as a function of the concentration gradient across the lipid bilayer, for different initial LUV radii.

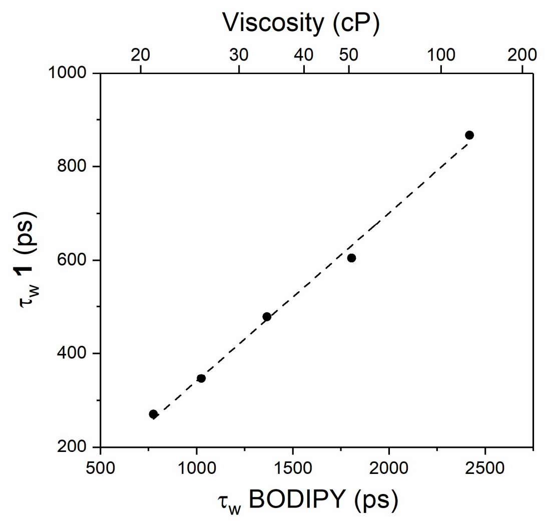

Fig. S16: Cross-calibration of the response of **1** externally added to DOPC LUVs with BODIPY molecular rotor [4].

Comment on SAXS and WAXS data analysis:

The acquired 2D SAXS and WAXS images were radially integrated to obtain the 1D scattering intensity diffraction patterns. The background (corresponding to a buffer-containing capillary) was then subtracted.

Fitting of the diffraction peaks was done semi-automatically with a custom-built MATLAB® script. The peaks were fitted to a single or double Voigt function (the later was used if it allowed a decrease of 50% of the fitting residual with respect to the single peak option) to obtain the peak(s) position, intensity and width.

Calculation of the bilayer thickness – d_{HH}:

Normal strain ε_z was calculated from the bilayer thickness ($2z_H$). This number was obtained from the calculated d-spacing and the peak intensities from the two first diffraction peaks of the SAXS spectra. First, the form factor corresponding to a given diffraction peak at position q was calculated taking into account the Lorentzian correction as:

$$F_k = \sqrt{I_k\, q_k^2}$$

Then, the headgroup position with respect to the center of the bilayer was then approximated using the method suggested by *Rappolt et al.*[5] as:

$$z_H = \pm \frac{d}{2\pi} \arccos\left(\frac{c1 - \sqrt{8(r_F c_3)^2 + 8(c_2 - r_F c_4)(r_F c_3) + c_1^2}}{4 r_F c_3} \right)$$

with:

$$r_F = \frac{F1}{F2};$$

$$c_1 = 2\sigma_H \exp\left[-2\left(\frac{\pi\sigma_H}{d}\right)^2\right]; \quad c_2 = -|\rho_r|\sigma_C \exp\left[-2\left(\frac{\pi\sigma_C}{d}\right)^2\right]; \quad c_3 = 2\sigma_H \exp\left[-8\left(\frac{\pi\sigma_H}{d}\right)^2\right]; \quad c_4 = -|\rho_r|\sigma_C \exp\left[-8\left(\frac{\pi\sigma_C}{d}\right)^2\right]$$

And where: σ_H = Gaussian distribution for the headgroup, σ_C = Gaussian distribution for the tails and ρ_r = Minimum to maximum electron density ratio. Values for these parameters were taken from the literature and are shown below:

**Parameter**	σ_H	σ_C	ρ_r
Value	3.1	4.4	0.9

As we are mostly interested in the change of d_{HH} with pressure, small deviations in bilayer thickness compared to the actual values would be acceptable.

In the case of DOPC at low pressures (<50 MPa), the 2nd order peak could not be clearly distinguished. However, plotting d_{HH} as a function of pressure in the range of 40-160 MPa resulted in a linear trend. By performing a linear fit on those points, we could extrapolate the values for the bilayer's thickness at lower pressures (Fig. S17).

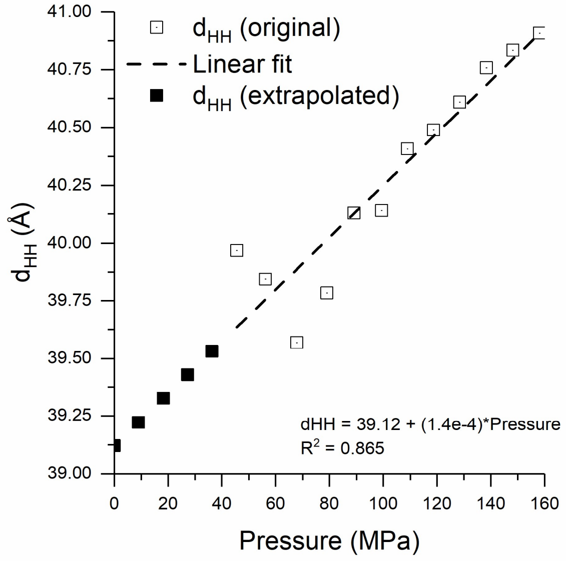

Fig. S17: Extrapolated values for DOPC d_{HH} at low pressures.

Calculation of the lipid area – A_L:

Calculation of the area per lipid differed between the gel DPPC and liquid disordered DOPC phases.

For the DPPC composition, the WAXS peaks were used. If two peaks could not be identified (d_{20} and d_{11}, see Fig. S7), the same value was given to both. Then, the area per lipid chain was computed using the standard formula for an orthorhombic packing[11] as:

$$A_C = \frac{a_c\, b_c}{2}$$

Where

$$a_c = 2d_{20} \quad b_c = \frac{d_{11}}{\sqrt{1 - \left(\frac{d_{11}}{2d_{20}}\right)^2}}$$

And finally, lipid area was approximated as[12]:

$$A_L = \frac{2A_C}{\cos(\beta)}$$

With β being the lipid tilt angle, which itself was approximated from the bilayer thickness d_{HH} as[11]:

$$\cos(\beta) = \frac{d_{HH}}{2N(1.27\text{Å}) + 2D_H}$$

Where N is the number of carbons in the lipid tail (16 for DPPC) and D_H represents the headgroup thickness (assumed 3.1 Å).

On the other hand, the area per chain (A_C) and area per lipid (A_L) for DOPC molecules were as suggested by *Mills et al.*[12]:

$$A_C = \frac{2}{\sqrt{3}}\left(\frac{2\pi}{q}\right)^2$$

$$A_L \cong 1.32\left(\frac{9\pi}{4q}\right)^2$$

Where q is the position of the WAXS peak.

Comparison between obtained and literature values:

Lipid	Parameter	Estimated	Literature
**DOPC**	d_{HH} (Å)	39.12	35.3[13]/38.42[14]
	Tilt angle β (°)	-	-
	A_C (Å^2)	23.4	23.7[12]
	A_L (Å^2)	67.72	71.8[12]/68.72[14]
**DPPC**	d_{HH} (Å)	43.28	42.8[11]
	Tilt angle β (°)	30.43	31.6[11]
	A_C (Å^2)	20.57	20.2[11]
	A_L (Å^2)	47.7	47.3[11]

As seen in the table above, our parameter estimates (at 0MPa) reasonably agree with reported values.

Discussion on effect of Poisson's ratio on negative compressibility:

The Poisson ratio was approximated from our SAXS/WAXS measurements by plotting ε_A (in-plane area strain) vs ε_z (normal strain) and finding the slope. The Poisson's ratio ν can then be extracted from the slope as suggested by Terzi et al.[15]:

$$\nu_{zx} := -\frac{\varepsilon_A}{2\varepsilon_z}$$

which yielded $\nu_{DOPC} = 0.32\pm0.03$ and $\nu_{DPPC} = -0.42\pm0.05$ respectively. It should be noted that these figures are for the response to uniform hydrostatic compression.

We note that while it is generally assumed that $\nu = 0.5$ for biological materials, direct experimental observation is challenging. In fact, theoretical work carried out by Jadidi et al.[16] suggested the Poisson ratio for fluid membranes in the *zx* direction was ~0.25, in reasonable agreement with our results.

We obtained a negative Poisson's ratio for membranes in the gel-phase under hydrostatic conditions, this would suggest DPPC membranes have a more uniform compressibility with compression occurring both in-plane and in the direction normal to the bilayer's surface. This behavior is consistent with the significantly increased lateral packing of the DPPC hydrocarbon chains relative to those in DOPC, resulting in a much lower in-plane compressibility which is closer to the membrane compressibility along the hydrocarbon chain axis.

We suggest the higher anisotropy of DOPC membranes structure (as compared to DPPC ones) allows them to sustain in-plane pressure-induced deformations. However, when DPPC is compressed, the membrane as a whole will buckle in response to the tighter chain packing.

Notes and references:

1 I. López-Duarte, P. Chairatana, Y. Wu, J. Pérez-Moreno, P. M. Bennett, J. E. Reeve, I. Boczarow, W. Kaluza, N. A. Hosny, S. D. Stranks, R. J. Nicholas, K. Clays, M. K. Kuimova and H. L. Anderson, *Org. Biomol. Chem.*, 2015, **13**, 3792–3802.

2 A. Vyšniauskas, I. López-Duarte, N. Duchemin, T. T. Vu, Y. Wu, E. M. Budynina, Y. A. Volkova, E. Peña Cabrera, D. E. Ramírez-Ornelas and M. K. Kuimova, *Phys. Chem. Chem. Phys.*, 2017, **19**, 25252–25259.

3 M. R. Dent, I. López-Duarte, C. J. Dickson, P. Chairatana, H. L. Anderson, I. R. Gould, D. Wylie, A. Vyšniauskas, N. J. Brooks and M. K. Kuimova, *Chem. Commun.*, 2016, **52**, 13269–13272.

4 M. R. Dent, I. López-Duarte, C. J. Dickson, N. D. Geoghegan, J. M. Cooper, I. R. Gould, R. Krams, J. A. Bull, N. J. Brooks and M. K. Kuimova, *Phys. Chem. Chem. Phys.*, 2015, **17**, 18393–18402.

5 M. Rappolt, *J. Appl. Phys.*, , DOI:10.1063/1.3393600.

6 J. Ferguson and A. W. H. Mau, *Aust. J. Chem.*, 1973, **26**, 1617–1624.

7 G. A. Vliegenthart and G. Gompper, *New J. Phys.*, 2011, **13**, 045020.

8 J. Lidmar, L. Mirny and D. R. Nelson, *Phys. Rev. E*, 2003, **68**, 051910.

9 A. Wright, *Nat. Phys.*, 2007, **3**, 19–19.

10 O.-Y. Zhong-can and W. Helfrich, *Phys. Rev. Lett.*, 1987, **59**, 2486–2488.

11 W. J. Sun, S. Tristram-Nagle, R. M. Suter and J. F. Nagle, *Biophys. J.*, 1996, **71**, 885–891.

12 T. T. Mills, G. E. S. Toombes, S. Tristram-Nagle, D. M. Smilgies, G. W. Feigenson and J. F. Nagle, *Biophys. J.*, 2008, **95**, 669–681.

13 S. Tristram-Nagle, H. I. Petrache and J. F. Nagle, *Biophys. J.*, 1998, **75**, 917–925.

14 W. Ding, M. Palaiokostas, G. Shahane, W. Wang and M. Orsi, *J. Phys. Chem. B*, 2017, **121**, 9597–9606.

15 M. M. Terzi, M. Deserno and J. F. Nagle, *Soft Matter*, 2019, **15**, 9085–9092.

16 T. Jadidi, H. Seyyed-Allaei, M. R. R. Tabar and A. Mashaghi, *Front. Bioeng. Biotechnol.*, 2014, **2**, 1–6.
